# An improved YOLO11 network integrated with sample generation for the intelligent detection of steel plate surface defects

**DOI:** 10.1371/journal.pone.0356877

**Published:** 2026-08-25

**Authors:** Luya Yang, Min Zhang, Yaxian Gao

**Affiliations:** School of Information Engineering, Shaanxi Xueqian Normal University, Xi’an, Shaanxi, China; Zhejiang Normal University, CHINA

## Abstract

To satisfy the stringent surface quality requirements imposed for industrial steel plates, automated defect detection techniques must balance high accuracy with low computational latency. In this paper, a lightweight detection method integrating adaptive image enhancement, generative sample synthesis, and an optimized detection model is proposed. First, an AW-ACE algorithm is introduced to resolve low contrast levels by dynamically merging color channels using information entropy. Second, an ES-DCGAN model based on ECA and an SELU is used to synthesize diverse and high-quality defect samples. Finally, we design a lightweight model, i.e., GE-YOLO11n, by incorporating GhostConv and ECA into YOLO11n to optimize the feature extraction process for small-scale defects. The entire framework yields a 1.5% mAP improvement and a 0.3 ms detector-only latency reduction relative to the baseline. Ablation studies demonstrate that the complete pipeline, comprising the ES-DCGAN, AW-ACE, and GE-YOLO11n modules, improves the overall mAP by 6.3% and reduces the number of required parameters by 21.4%. Compared with the evaluated models, our proposed method achieves an mAP value of 91.3% and a full-pipeline latency level of 7.4 ms, outperforming the other compared models under the experimental conditions specified in this study. This method delivers a highly efficient and robust solution for attaining real-time industrial quality control.

## 1. Introduction

As fundamental materials in aerospace, automotive manufacturing, and bridge construction scenarios, steel plates demand rigorous surface quality assurances. Even minor surface defects, including crazing, inclusions, and scratches, can critically compromise the mechanical integrity and long-term operational safety of finished products [1]. Concurrently, China’s 14th Five-Year Plan for the Development of Intelligent Manufacturing underscores the strategic imperative of advancing intelligent detection equipment, with an explicit emphasis on digital noncontact precision measurement technologies. This policy framework has galvanized substantial collaborative momentum among enterprises, universities, and research institutions, fostering an environment that enables technological innovations and instrumentation development processes. Against this backdrop, the design and implementation of high-efficiency, high-accuracy intelligent inspection methods for steel plate defects have emerged not only as pressing industrial necessities but also as a pivotal research avenue that directly aligns with the national strategic directives.

The evolution of defect detection technologies for steel plates can be broadly categorized into three successive stages. Initially, manual inspection was the predominant approach, but it suffers from inherently low detection accuracy, inadequate real-time performance, and the substantial consumption of both human and material resources. Conventional nondestructive testing techniques, such as eddy current testing, infrared thermography, and magnetic flux leakage testing [2], were subsequently introduced, yielding incremental improvements. For instance, Yoshimura [3] developed a noncontact system based on low-frequency eddy current testing for detecting slit defects on the reverse sides of steel plates. Nevertheless, these methods exhibit a limited ability to identify complex surface irregularities, primarily because of their sensitivity to environmental noise and reliance on predetermined feature parameters. In recent years, the rapid advancement of deep learning has led to remarkable breakthroughs in computer vision [4], and its integration with industrial vision systems has enabled the automated identification, classification, and localization of defects in steel plate production scenarios. Chigateri [5] proposed a periodic defect detection framework that extracts defect image features via a convolutional neural network (CNN), subsequently feeding them into a long short-term memory (LSTM) network for recognition purposes. Zhang [6] employed an R-CNN baseline model and enhanced visual features by incorporating an attention mechanism with a graph convolutional neural network. Song [7] introduced an improved Faster R-CNN architecture with deformable convolution capabilities to improve the detection performance achieved for large-scale defects concerning intricate geometries. Liu G [8] developed an enhanced object detector, DLF-YOLOF, which adopts an anchor-free design to reduce the number of required hyperparameters and integrates deformable convolutional networks along with a local spatial attention module into its feature extraction backbone. Focusing on hot-rolled strip steel, Mi [9] proposed a surface defect detection method that applies automatic gamma correction and Otsu thresholding to eliminate redundant background interference and incorporates a dedicated small-defect detection layer into the YOLO framework to improve its sensitivity to small-scale defects.

Despite the notable progress achieved in steel plate defect detection, the existing studies have predominantly relied on public benchmark datasets and concentrated on isolated detection model improvements, with their innovations often limited to superficial modifications of individual modules. Crucially, these approaches lack a systematic framework that is tailored to industrial environments. In this context, three critical deficiencies persist. First, the current methods are heavily customized to handle specific defect types or fixed illumination conditions and lack adaptive mechanisms to address coexisting defects and fluctuating lighting conditions. In our study, we introduce an adaptive feature enhancement module, which leverages multiscale extraction and normalized fusion techniques to accommodate diverse defect classes and illumination variations. Second, generative adversarial networks suffer from modal collapse and insufficient fidelity, especially for small-scale or low-contrast defects. We incorporate an efficient channel attention mechanism into both the generator and the discriminator to reinforce the focus on defect-relevant features, thereby improving the realism and diversity of the generated samples. Third, lightweight YOLO variants tend to sacrifice fine-grained feature capture capabilities for efficiency, undermining the detection of small-scale defects. We redesign the backbone and head with a lightweight feature fusion module that enhances small-defect representations while preserving high computational efficiency, thus reconciling accuracy and efficiency.

To systematically address these challenges, a defect detection methodology encompassing three coordinated innovations is developed herein: an adaptive image enhancement process, an attention-augmented data augmentation scheme, and an optimized lightweight detection architecture. This integrated solution differentiates our work from previous efforts and offers a viable pathway for industrial deployment.

## 2. Overall research framework

**Fig 1:**
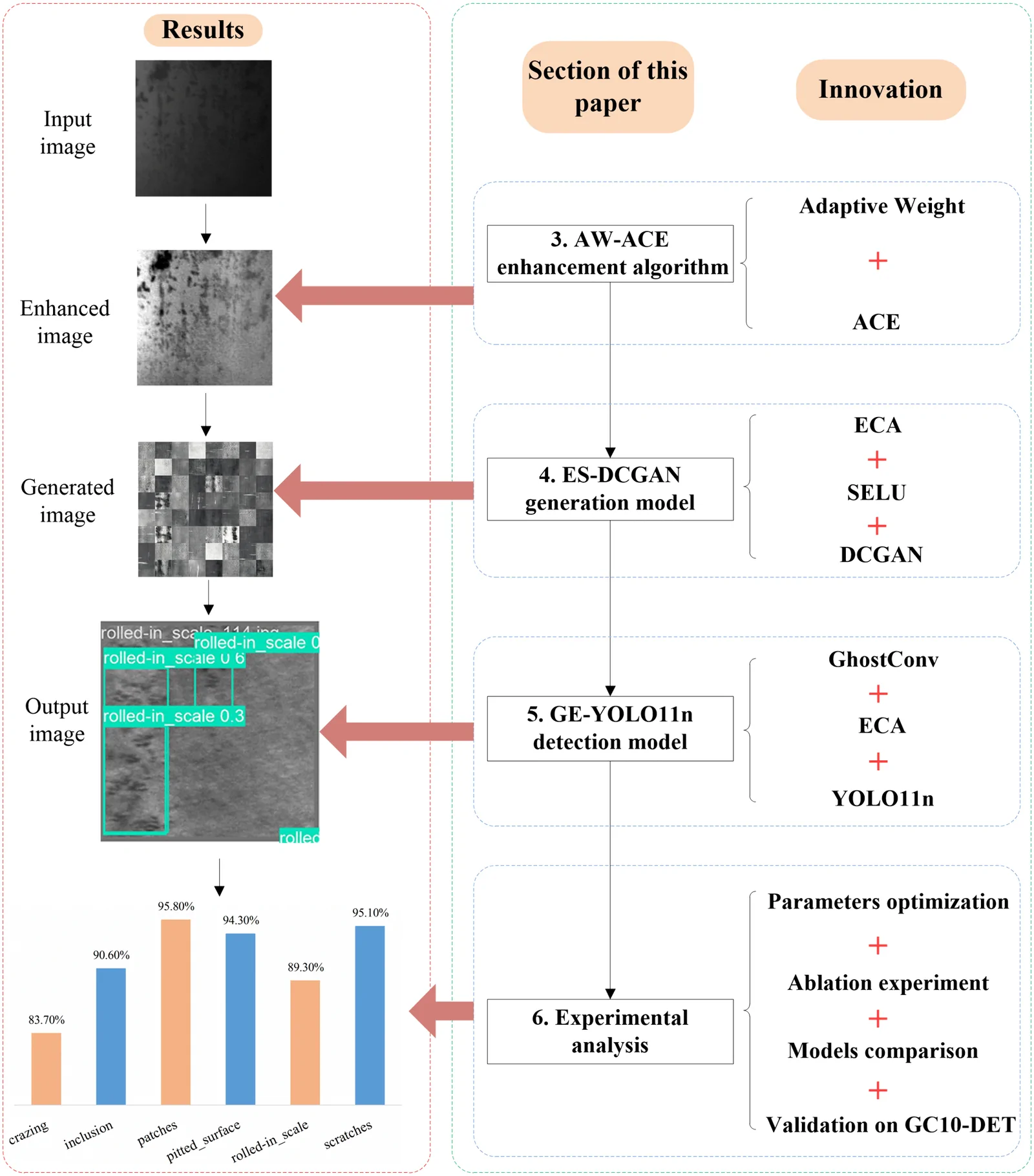
Research framework.

The methodological framework and research strategies used across the sections of this work are presented in Fig 1, which comprises four parts. Section 3 presents the defect image preprocessing scheme, which enhances the traditional ACE algorithm by incorporating an adaptive weight calculation method. Section 4 introduces the ES-DCGAN, a generative model for defect sample augmentation that integrates the ECA mechanism and SELU activation function into the DCGAN architecture. Section 5 details the lightweight GE-YOLO11n detection model, which embeds GhostConv and ECA modules into YOLO11n to improve its efficiency and accuracy. Section 6 reports the conducted experimental evaluations, covering hyperparameter optimization work, ablation studies, and comparative analyses against state-of-the-art alternatives.

## 3. Defect image preprocessing: The AW-ACE enhancement algorithm

Steel plate surfaces frequently contain subtle defects such as crazing and scratches, which are often poorly distinguishable in raw images. Such low contrast levels impair the reliability of automated detection and analysis methods. To address this, an effective image enhancement scheme is essential for accentuating these minute details and producing high-fidelity inputs for downstream processing tasks.

The automatic color enhancement (ACE) algorithm [10] enhances image contrast levels by exploiting the spatial relationships between color and intensity across different pixels. A key limitation of this approach is that the weights *W*_*k*_ employed for the R, G, and B channels are empirically fixed, making the process susceptible to subjective judgments. We therefore present AW-ACE, an extension that introduces adaptive weighting to address this drawback. The performances of the different methods are compared in Table 1. Unlike one-dimensional entropy, variance, gradients, and correlation coefficients, two-dimensional entropy accounts for both pixel grayscale distributions and spatial neighborhood structures. It offers robust noise suppression, effective detail preservation, and adaptive weight assignment capabilities and eliminates manual parameter tuning requirements. Consequently, the fused image provides richer information and enhanced layering effects, rendering it highly suitable for high‑precision applications such as medical imaging and industrial inspections.

**Table 1:** Comparison among the common weight calculation methods.

Method	Advantage	Disadvantage	Applicable scenarios
Fixed weight	• Fast calculation process • Simplicity	Not adaptive	• Grayscale conversion of ordinary images • Real-time video processing
One-dimensional entropy	• Adaptability • Information retention	Lacks spatial structure perception capabilities	• Images with obvious contrast differences and low noise levels
Variance/Standard deviation	• Simple calculation p proc	Sensitive to noise and prone to weight biases	• Low-contrast cases • Clear and noise-free images
Gradient	• Good edge retention capabilities • Clear details	Poor noise resistance and instability in low-light images	• Industrial inspections • Images requiring edge preservation
Correlation coefficient	• Reduces the degree of information overlap • Maximizes the total amount of information	High computational complexity and slow speed	• Medical imaging • Multimodal fusion
Two-dimensional entropy	• Takes both grayscale and spatial structures into account • Distinguishes between details and noise • Self-adaptation	Requires a certain number of computations	• Medicine and remote sensing • Industrial high-precision detection

(1) Acquiring reconstructed images in the spatial domain

The R, G, and B channels are processed separately, and the spatial domain is adjusted to complete the color correction process and obtain the reconstructed image. This operation is formalized as follows:


$$\begin{array}{c} R_{c}=\sum_{j\in subset,j\neq p}\frac{r\left(I_{c}\left(p\right)-I_{c}\left(j\right)\right)}{d\left(p,j\right)} \end{array}$$ (1)

where the range of the subset is the 7 × 7 neighborhood of p. $I_{c}\left(p\right)-I_{c}\left(j\right)$ is the difference in brightness between *p* and *j*. d(p,j) is the distance metric function defined as follows:


$$\begin{array}{c} d\left(p,j\right)=\sqrt{{\left(x_{p}-x_{j}\right)}^{\text{2}}+{\left(y_{p}-y_{j}\right)}^{\text{2}}} \end{array}$$ (2)

where $x_{p}$, $y_{p}$ and $x_{j}$, $y_{j}$ are the horizontal and vertical coordinate values of *p* and *j*, respectively.

The brightness representation function *r* (*) is as follows:


$$\begin{array}{c} r\left(t\right)=\left\{ \begin{array}{c} @l1\;\;\;\;\;\;\;\;\;\;\;\;\;t\;<-T\;\\ t/T\;\;\;\;\;-T\leq t\leq T\\ -1\;\;\;\;\;\;\;\;\;\;\;\;t\;>\;T\; \end{array}\right. \end{array}$$ (3)

The threshold *T* is defined as the 70th percentile of the absolute differences computed within the 7 × 7 local region:


$$\begin{array}{c} T=percentile\left(\left|t\right|,70\%\right) \end{array}$$ (4)

(2) Dynamic image expansion

ACE is applied to single-channel images. For color images, channel separation is performed first, and the corresponding linear extension is given by


$$\begin{array}{c} L\left(x\right)=\frac{R\left(x\right)-minR}{maxR-minR} \end{array}$$ (5)

For each enhanced channel R(x), we calculate its minimum and maximum values and then apply linear normalization to map the channel to [0,1].

(3) Adaptive weight calculation

Two-dimensional entropy [11] is introduced to automatically determine the three-channel merging weights. The weights are derived from the binary (*m*,*n*) formed by the pixel grayscale and its 3 × 3 neighborhood mean. For boundary pixels, we apply mirror-symmetric padding to extend the image (e.g., for pixels in the topmost row, their upper-side neighbors match their lower-side adjacent pixels). This strategy preserves local grayscale continuity and avoids the edge artifacts that are typically caused by zero-filling or replication operations. The proportion *P*_*mn*_ can be obtained as follows:


$$\begin{array}{c} P_{mn}=F\left(m,n\right)/N \end{array}$$ (6)

where *F*(*m*, *n*) is the frequency of (*m*, *n*) and *N* is the total number of pixels.

The two-dimensional entropy metric is calculated as follows:


$$\begin{array}{c} E=\text{-}\sum_{m=0}^{255}\sum_{n=0}^{255}P_{mn}logP_{mn} \end{array}$$ (7)

**Fig 2:**
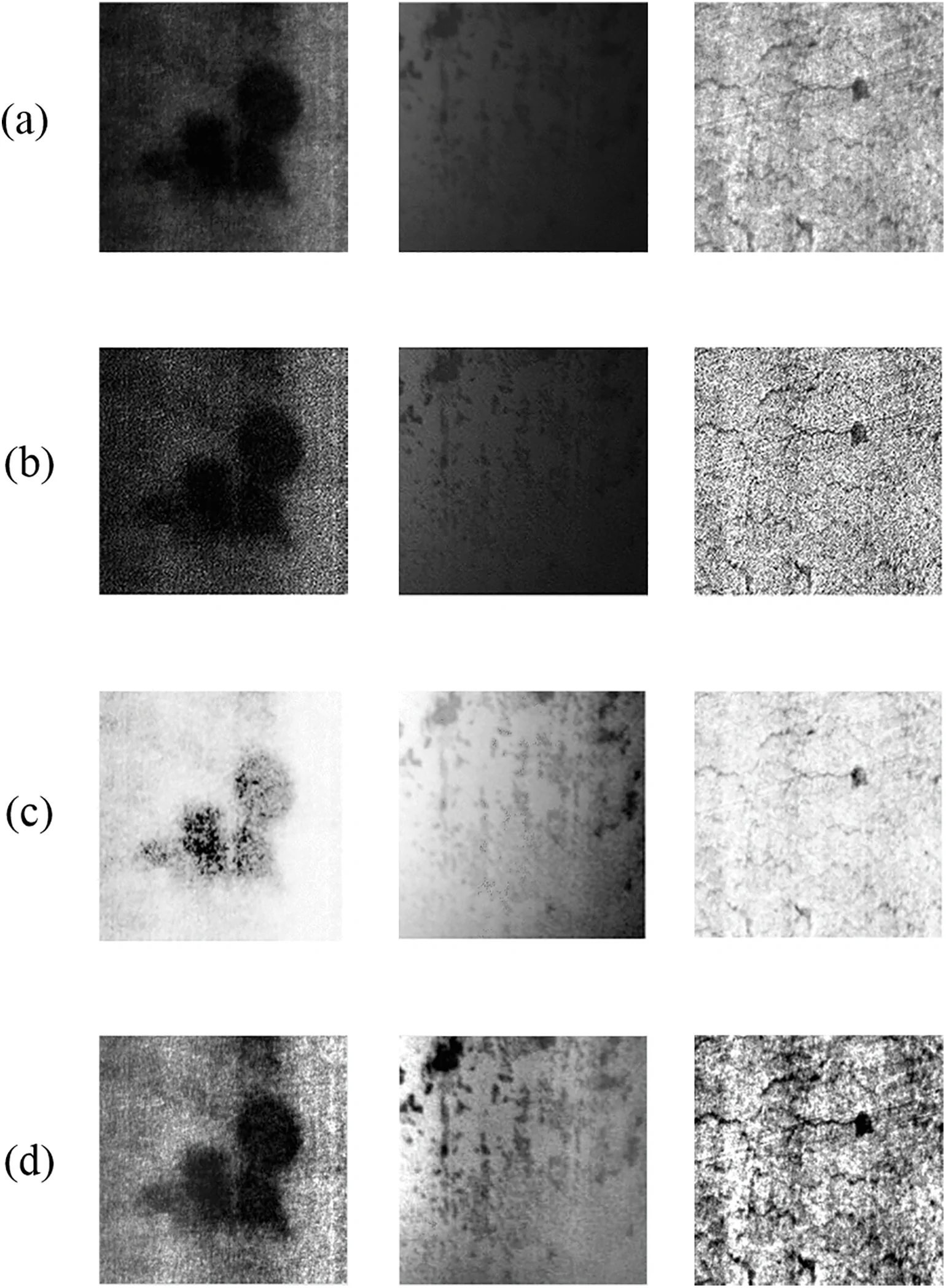
Processing results yielded by different enhancement algorithms. (a) Original images. (b) Laplacian-enhanced images. (c) SSR-enhanced images. (d) AW-ACE (the method of this paper) images.

The merging weight for each channel can be calculated from its two-dimensional entropy ratio as shown below:


$$\begin{array}{c} W_{k}=\frac{E_{k}}{\sum_{l=1}^{\text{3}}E_{l}} \end{array}$$ (8)

where *E*_*k*_ is the two-dimensional entropy of channel *k*. The three-channel images are merged to yield an AW-ACE-enhanced image. The original images utilized in this study are sourced from the NEU-DET dataset, as released by Northeastern University [12,13]. The partial visualization results obtained from various enhancement algorithms are shown in Fig 2. The Laplacian algorithm [14] yields images with low contrast levels and substantial noise interference. Moreover, the SSR algorithm [15,16] results in the omission of certain defect features, with the remaining features exhibiting poor visibility. In contrast, the AW-ACE algorithm demonstrates superior performance, with its results characterized by pronounced defect features, high contrast levels, and effective defect enhancements, while maintaining an average processing time of 1.2 ms per image.

**Fig 3:**
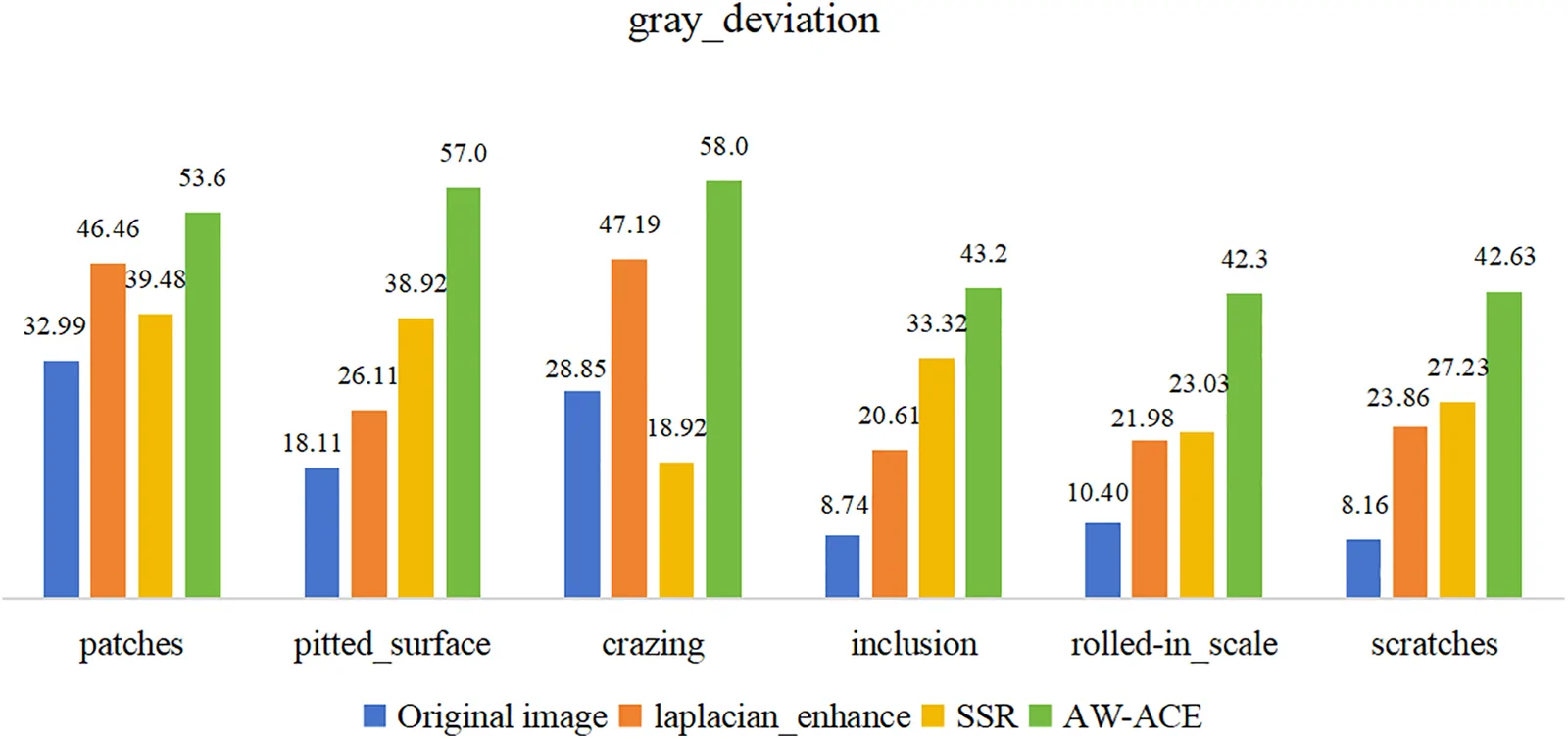
Gray_deviation extraction results produced for the original and enhanced images.

**Fig 4:**
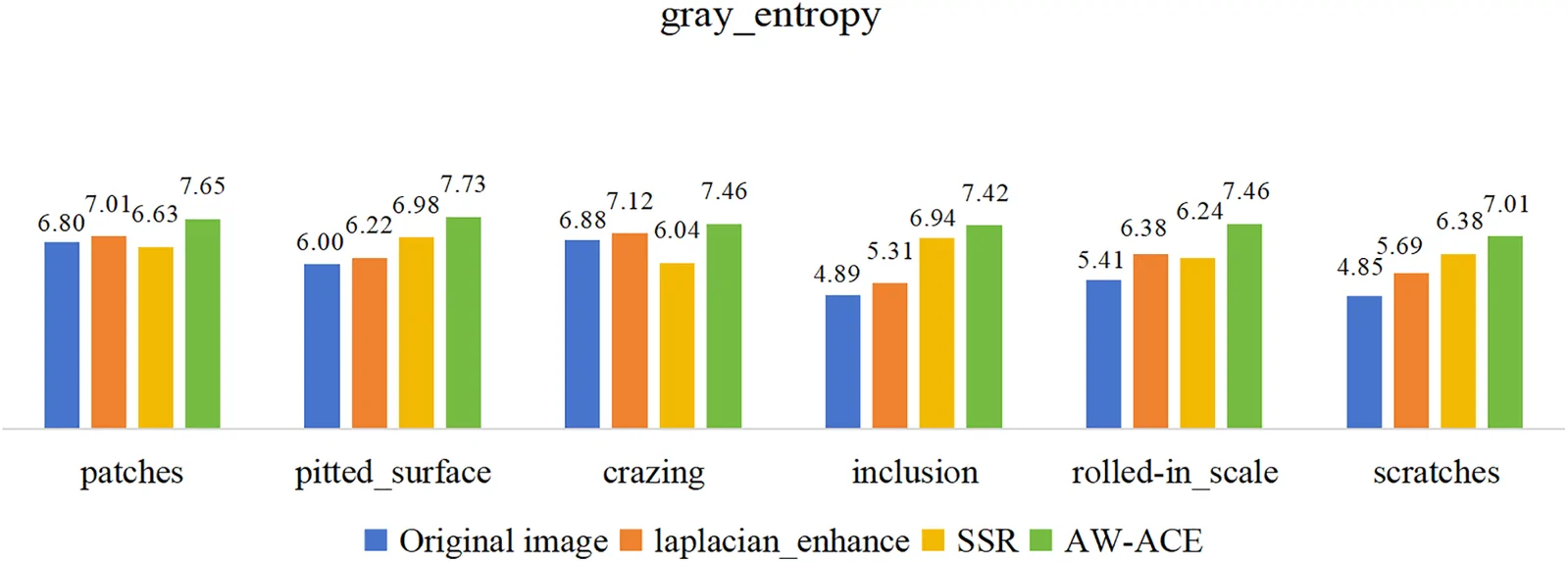
Gray_entropy extraction results produced for the original and enhanced images.

Gray_deviation and gray_entropy are adopted as the image quality evaluation metrics. Specifically, gray_deviation quantifies the fluctuation degrees exhibited by grayscale values, whereas gray_entropy measures the image information content, reflecting the randomness or uncertainty of the grayscale distribution. These two metrics are extracted from both the original and enhanced images for the six defect classes, and the corresponding results are shown in Figs 3 and 4. All three enhancement methods improve the gray_deviation and gray_entropy values relative to those of the original images. Among them, the AW-ACE algorithm yields the highest values for both metrics, indicating substantial grayscale variations, a nonuniform grayscale distribution, and richer textures and detailed information in the enhanced images.

## 4. Defect sample expansion: The ES-DCGAN defect image enhancement algorithm

The traditional generative adversarial networks (GANs) are confronted with inherent challenges such as modal collapse and gradient vanishing during their training processes. To mitigate these issues, the deep convolutional GAN (DCGAN) incorporates convolutional architectures, transposed convolution operations, batch normalization operations, and LeakyReLU activation functions. In the context of industrial manufacturing, the DCGAN has been applied to generate steel plate defect images, thereby facilitating defect image analyses and diagnostic model training processes while effectively alleviating the scarcity of defective samples in production cases. In this paper, we propose an improved ES-DCGAN model, which integrates the efficient channel attention (ECA) mechanism and the scaled exponential linear unit (SELU) activation function into the DCGAN framework to further enhance its generation performance.

### 4.1. ECA mechanism

Attention mechanisms are crucial for improving deep learning models because they emphasize relevant features. However, the squeeze-and-excitation (SE) module incurs extra computational costs through its channel dimensionality reduction and subsequent restoration operations. The ECA [17] avoids such redundant operations, offering lower parameter overhead. Given this efficiency advantage, we adopt ECA over other prevalent methods (e.g., SE, the CBAM, and CA) for our framework. First, ECA introduces the fewest additional parameters and the least computational overhead, which is critical for maintaining a stable adversarial training process in the DCGAN. In contrast, SE adds two fully connected layers, the CBAM incorporates an extra spatial attention branch, and CA involves performing encoding and fusion along two directional axes. Second, the ECA mechanism possesses an extremely simple architecture, consisting solely of global average pooling, a one-dimensional convolution, and a sigmoid activation—without any branches or complex dimensional transformations. This simplicity facilitates the symmetric embedding of this mechanism into the generator and the discriminator of the DCGAN, both of which are composed of stacked convolutional blocks. Third, ECA focuses exclusively on channelwise recalibration and deliberately avoids any spatial position operations, thereby preventing the generator from overrelying on spatial attention, which could otherwise introduce artifacts. Moreover, the CBAM adds spatial attention that may bias the generator, and CA embeds coordinate information that implicitly encodes spatial positions into channel weights, potentially altering the learning preferences of the generator. Therefore, ECA provides a clean and efficient channel-only attention mechanism that best suits our DCGAN-based generation task.

**Fig 5:**
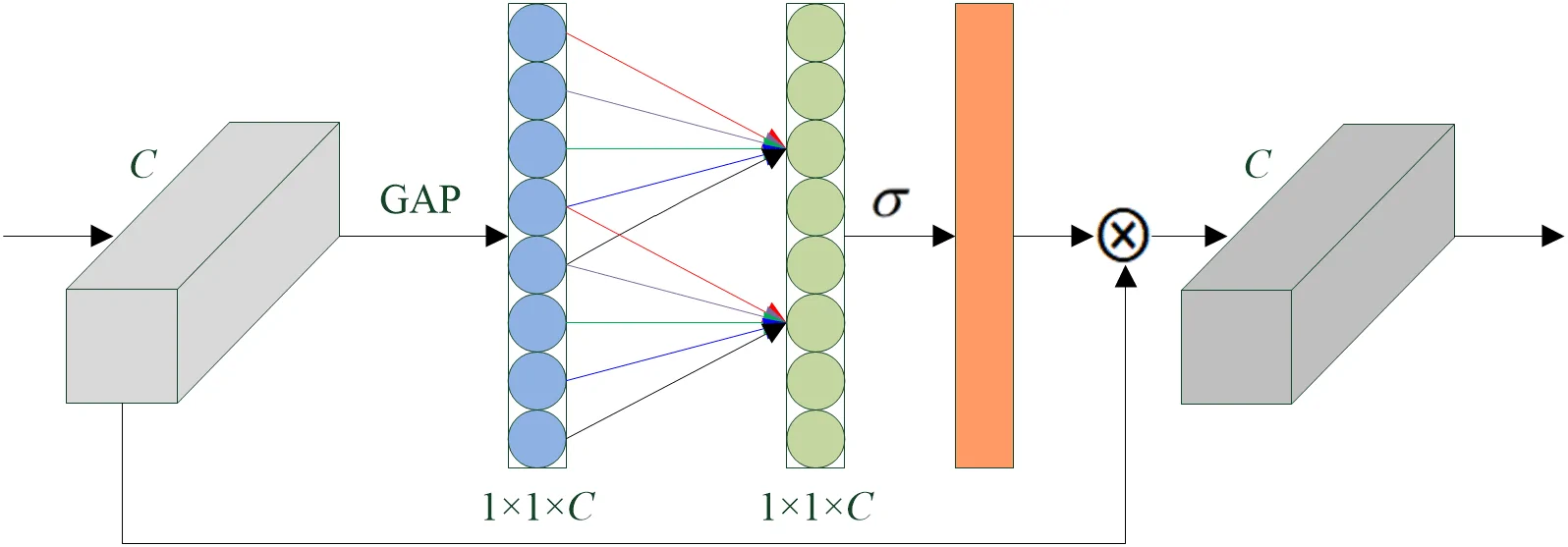
Schematic diagram of the ECA algorithm.


The ECA module, which uses local cross-channel interactions to efficiently capture dependencies and increase the expressiveness of the constructed model, is shown in Fig 5. This module performs global average pooling, followed by a 1D convolution (with a kernel size of *k*) and a sigmoid activation function to produce weights *ω*. The associated formula is as follows:

$$\begin{array}{c} \omega =\sigma \left(C1D_{k}\left(y\right)\right) \end{array}$$ (9)

where *C*1*D*_*k*_ is a 1-dimensional convolution operation and *σ* is the sigmoid function.

Finally, the ECA module multiplies the obtained weights with the corresponding elements of the original input feature map in an elementwise manner to produce the final output feature map.

### 4.2. SELU activation function

The scaled exponential linear unit (SELU) [18], which is an improved variant of the ELU, introduces an automatic normalization mechanism that drives activations toward zero means and unit variances. Unlike the ReLU and LeakyReLU, which cannot regulate the activation distribution, or the ELU, which only approximates zero means without enforcing unit variances, and in contrast with the Tanh and Sigmoid functions, which suffer from saturation-induced gradient vanishing issues, the SELU uniquely ensures stable propagation through deep networks. This property effectively mitigates the gradient vanishing and explosion problems, thereby enhancing the training stability of the DCGAN model. The SELU is formulated as follows:


$$\begin{array}{c} f\left(x\right)=\lambda \cdot \left\{ \begin{array}{c} @lx\;x>0\\ \alpha \left(e^{x}-1\right)\;x\leq 0 \end{array}\right. \end{array}$$ (10)

where *λ* and *α* are usually set to 1.0507 and 1.67326, respectively.

### 4.3. ES-DCGAN structure

**Fig 6:**
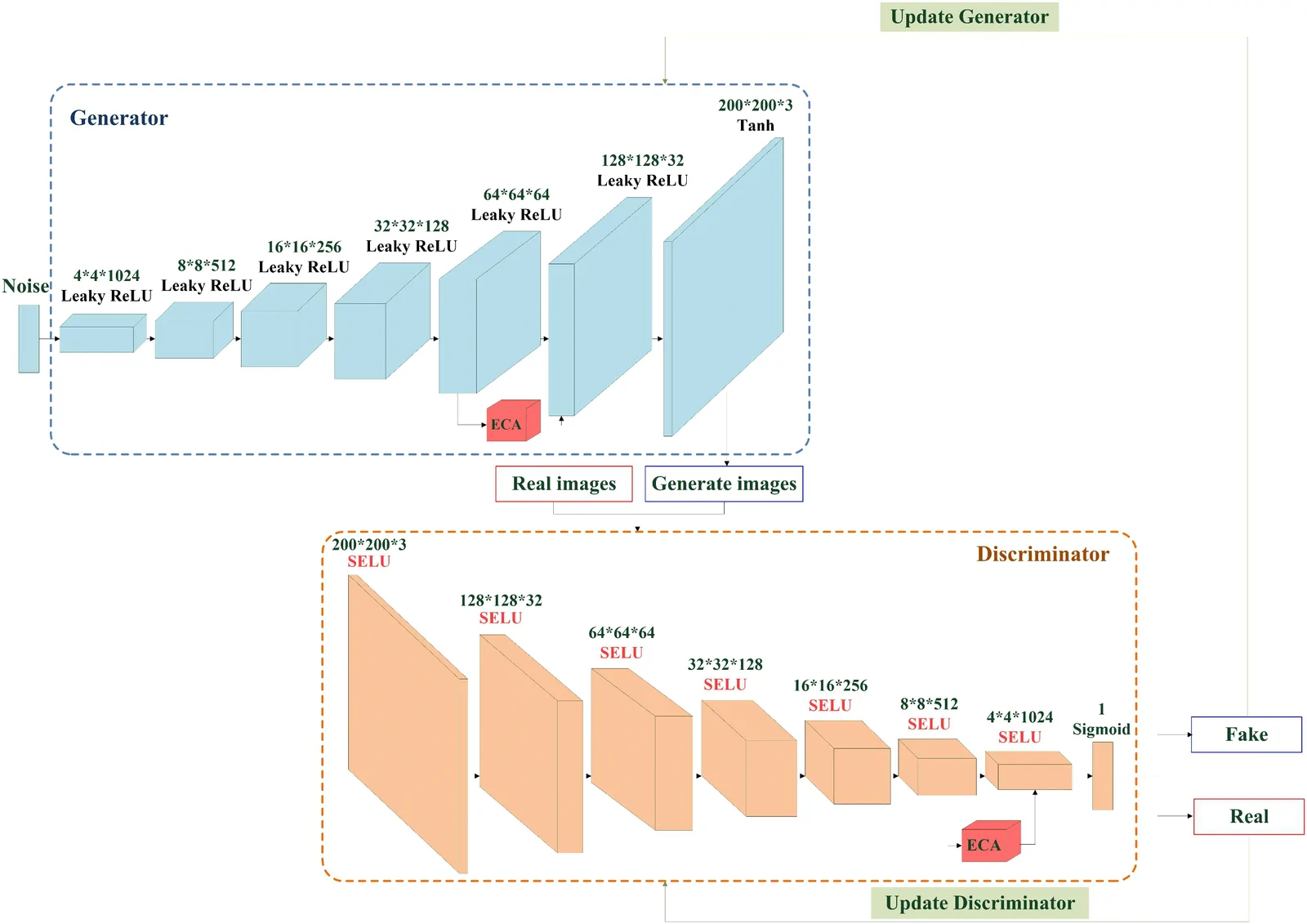
Network structure of the ES-DCGAN.

The proposed ES-DCGAN follows the standard DCGAN architecture, which consists of a generator and a discriminator [19,20]. The generator synthesizes realistic images from random noise, while the discriminator is trained to distinguish generated fake samples from real samples; the generator then iteratively improves its outputs based on the feedback received from the discriminator to deceive it. The overall network structure is illustrated in Fig 6. In our improved framework, the ECA mechanism is embedded into the generator to enhance its ability to address diverse feature information during the image synthesis process. For the discriminator, we incorporate both the ECA mechanism and the SELU activation function. Specifically, the original LeakyReLU function is replaced by an SELU in all layers except the output layer, which retains the sigmoid function. This design further improves the perceptual quality of the generated images.

(1) Loss function of the generator

$$\begin{array}{c} L_{G}=-E_{z\sim p\left(z\right)}\left[log\left(D\left(G\left(z\right)\right)\right)\right] \end{array}$$ (11)

where *G*(*z*) is the fake image output by the generator, *D*(*G(z*)) is the probabilistic prediction produced by the discriminator for the fake image, and the generator maximizes the probability of discriminator misjudgments by minimizing *L*_*G*_.

(2) Loss function of the discriminator

The formula for the loss function of the discriminator for real images is as follows:


$$\begin{array}{c} L_{D_{real}}=-E_{x\sim p_{data}\left(x\right)}\left[log\left(D\left(x\right)\right)\right] \end{array}$$ (12)

where *x* is the real image and *D*(*x*) is the probabilistic prediction produced by the discriminator for the real image.

The loss function of the discriminator for the generated images is as follows:


$$\begin{array}{c} L_{D_{fake}}=-E_{z\sim p\left(z\right)}\left[log\left(1-D\left(G\left(z\right)\right)\right)\right] \end{array}$$ (13)

where *G*(*z*) is the fake image output by the generator and *D*(*G*(*z*)) is the probabilistic prediction produced by the discriminator for the fake image.

Therefore, the loss function of the discriminator is as follows:


$$\begin{array}{c} L_{D}=L_{D_{real}}+L_{D_{fake}} \end{array}$$ (14)

The training hyperparameters are summarized in Table 2 and include a latent vector of 100, the Adam optimizer (with a learning rate of 0.0002), a batch size of 32, an epoch count of 150, and a random seed of 42. The specific structural parameters of the generator and discriminator are presented in Tables 3 and 4, respectively, which correspond to the network design shown in Fig 6.

**Table 2:** Training hyperparameter settings.

Parameter	Optimizer	Learning rate	β_1_/β_2_	Batch size	Number of epochs	Random seed
Value	Adam	0.0002	0.5/0.999	32	150	42

**Table 3:** Generator parameter settings.

Sequence number	Input size	Output size	Convolution kernel/step size	Activation function	Attention mechanism
1	100 × 1	4 × 4 × 1024	Transposed convolution, 4 × 4, stride = 1	LeakyReLU (α = 0.2)	
2	4 × 4 × 1024	8 × 8 × 512	Transposed convolution, 4 × 4, stride = 2	LeakyReLU (α = 0.2)	
3	8 × 8 × 512	16 × 16 × 256	Transposed convolution, 4 × 4, stride = 2	LeakyReLU (α = 0.2)	
4	16 × 16 × 256	32 × 32 × 128	Transposed convolution, 4 × 4, stride = 2	LeakyReLU (α = 0.2)	
5	32 × 32 × 128	64 × 64 × 64	Transposed convolution, 4 × 4, stride = 2	LeakyReLU (α = 0.2)	ECA
6	64 × 64 × 64	128 × 128 × 32	Transposed convolution, 4 × 4, stride = 2	LeakyReLU (α = 0.2)	
7	128 × 128 × 32	200 × 200 × 3	Transposed convolution, 4 × 4, stride = 2	Tanh	

**Table 4:** Discriminator parameter settings.

Sequence number	Input size	Output size	Convolution kernel/step size	Activation function	Attention mechanism
1	200 × 200 × 3	128 × 128 × 32	Convolution, 4 × 4, stride = 2	SELU	
2	128 × 128 × 32	64 × 64 × 64	Convolution, 4 × 4, stride = 2	SELU	
3	64 × 64 × 64	32 × 32 × 128	Convolution, 4 × 4, stride = 2	SELU	
4	32 × 32 × 128	16 × 16 × 256	Convolution, 4 × 4, stride = 2	SELU	
5	16 × 16 × 256	8 × 8 × 512	Convolution, 4 × 4, stride = 2	SELU	ECA
6	8 × 8 × 512	4 × 4 × 1024	Convolution, 4 × 4, stride = 2	SELU	
7	4 × 4 × 1024	1 × 1 × 1	Convolution, 4 × 4, stride = 1	Sigmoid	

**Fig 7:**
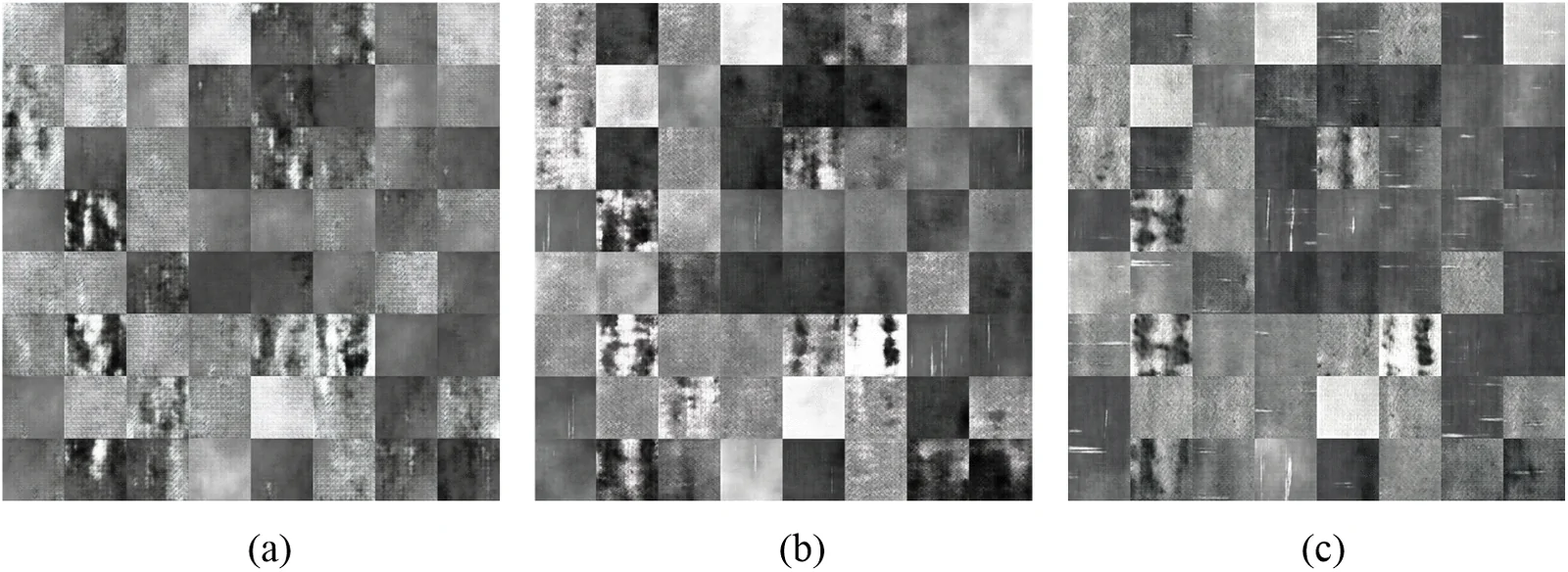
Steel plate defect images generated by the ES-DCGAN. (a) Epoch = 50. (b) Epoch = 100. (c) Epoch = 150.

The samples generated at the successive training stages are shown in Fig 7. After 50 epochs, only limited defect features are discernible, with most outputs remaining noisy and lacking clear structures. After 100 epochs, blurred defect textures gradually become visible. By 150 epochs, the generation performance stabilizes, producing defect regions that are clearly delineated and visually consistent in terms of their details.

**Fig 8:**
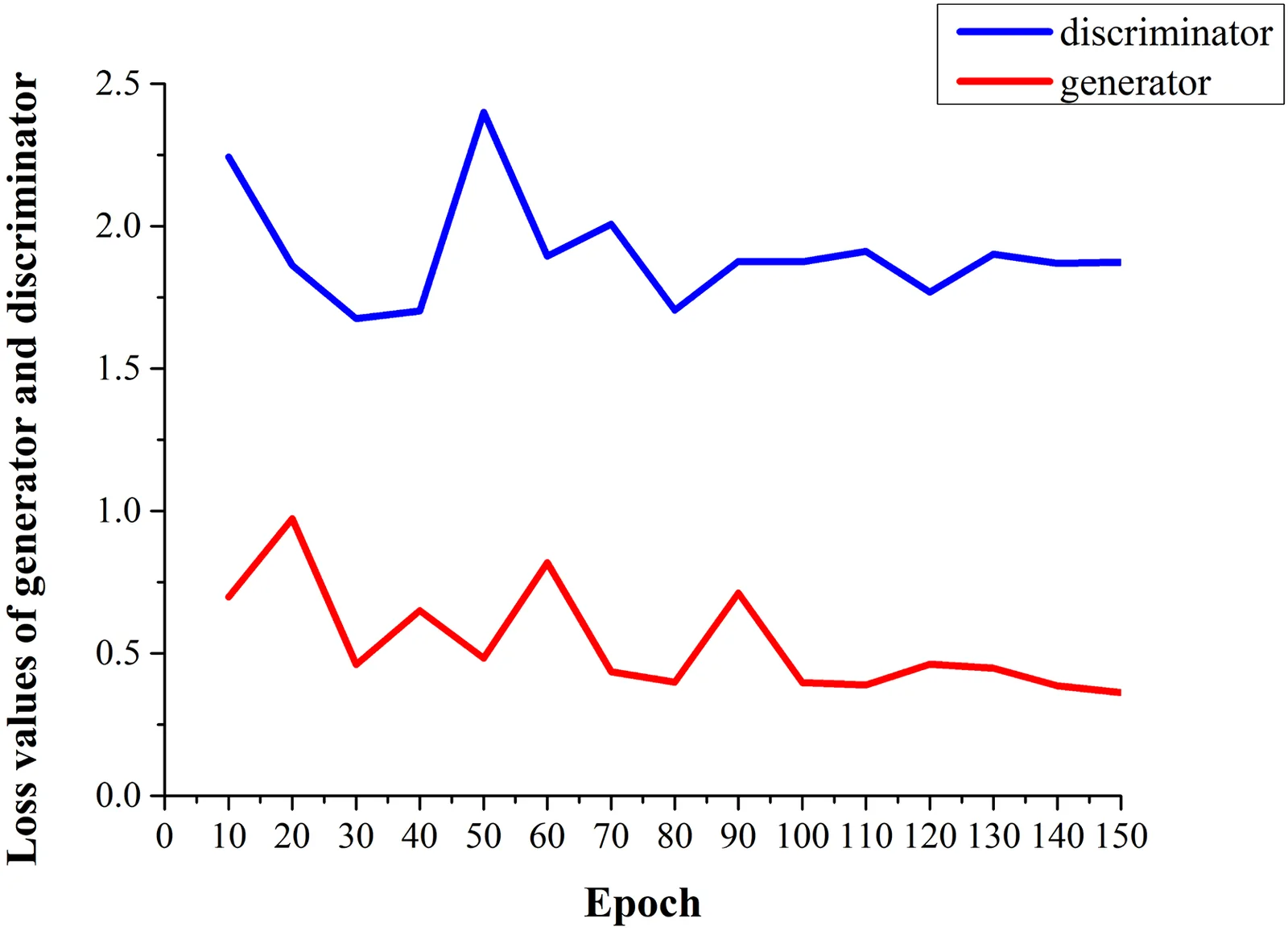
Loss values of the discriminator and generator.

The training losses are logged every 10 epochs. As shown in Fig 8, both the generator and discriminator losses converge and stabilize after 150 epochs, confirming that the generated images satisfy the desired quality standards.

The original NEU-DET dataset is split into training, validation, and test sets at a 7:2:1 ratio. Only the 1260 training images are used for sample generation purposes, resulting in a total of 3060 training samples (1260 original and 1800 synthesized samples). Pixel-based and feature-based distances are calculated to evaluate the diversity and memorization of the generated images. For pixel-based distance metrics, the Euclidean distance is evaluated in a pixelwise manner. For feature-based metrics, a 512-dimensional feature vector is extracted from each image using the conv4_4 layer of VGG19, and the Euclidean distance is subsequently computed between the resulting vectors. The formal definitions are provided as follows.

**Fig 9:**
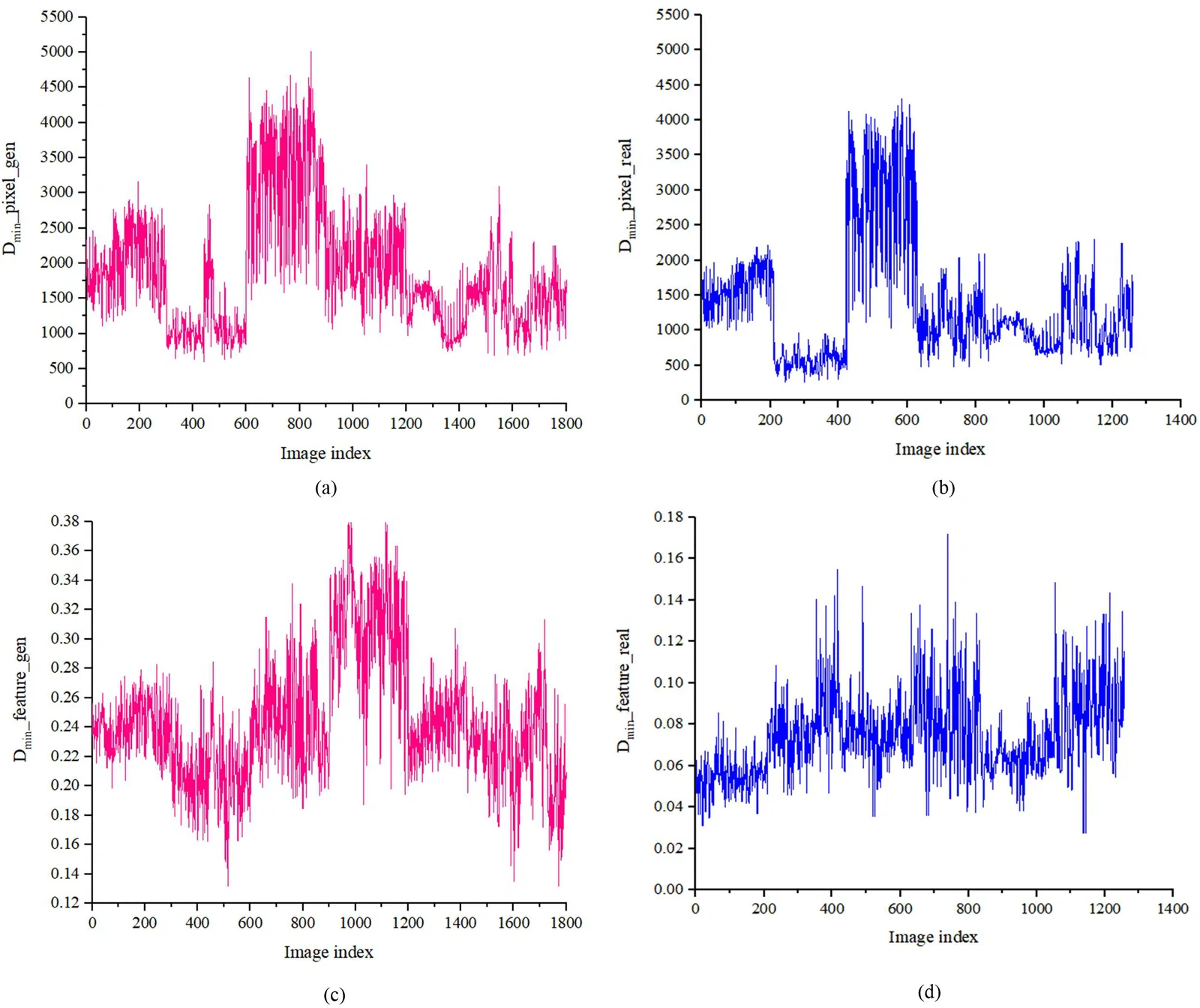
Minimum distance between generated and real images. (a) Minimum pixelwise distance between generated and real images. (b) Minimum pixelwise distance between pairs of real images. (c) Minimum feature distance between generated and real images. (d) Minimum feature distance between pairs of real images.

(1) Pixel-based distance: For each of the 1800 generated images, the minimum pixelwise distance to any of the 1260 real images is calculated and the D_min__pixel_gen distribution is shown in Fig 9(a). For each of the 1260 real images, the minimum pixelwise distance to all the other training images (excluding itself) is computed, the D_min__pixel_real is presented in Fig 9(b).
(2) Feature-based distance: For each of the 1800 generated images, the minimum feature distance to any of the 1260 real images is calculated and the D_min__feature_gen distribution is shown in Fig 9(c). For each of the 1260 real images, the minimum feature-based distance to all other training images (excluding itself) is computed, the D_min__feature_real (baseline) is presented in Fig 9(d).

**Fig 10:**
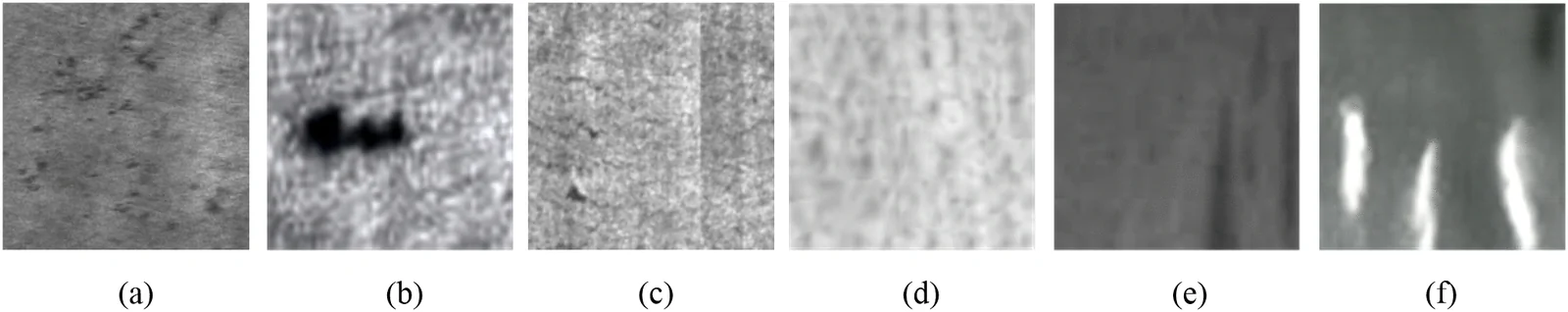
Per-class generation results. (a) Rolled-in scale. (b) Patches. (c) Crazing. (d) Pitted surface. (e) Inclusion. (f) Scratches.

The two distributions exhibit a high degree of overlap, and the distances in Fig 9(a) and Fig 9(c) are generally greater than those in Fig 9(b) and Fig 9(d), indicating that the generated images are diverse and are not near-duplicates of the real training images. Finally, Fig 10 displays the defect images generated for six defect classes.

### 4.4. Ablation experiment concerning the ES-DCGAN

To evaluate the effectiveness of each proposed component, we adopt the inception score (IS) and Fréchet inception distance (FID) as quantitative metrics for assessing the quality and diversity of the generated defect images. Ablation studies are conducted, and the results are summarized in Table 5. Specifically, the IS measures the clarity and diversity of the generated samples, whereas the FID quantifies the distributional discrepancy between the generated and real defect images. The experimental results demonstrate that the proposed ES-DCGAN achieves the highest IS and the lowest FID among all the compared models, indicating enhanced clarity, greater diversity, and closer distribution alignment with the real samples.

**Table 5:** Parameter comparison among different DCGAN variants.

Models	IS	FID	Single-image generation latency (ms)
DCGAN (baseline)	6.83	38.65	18.5
DCGAN+SE	7.58	32.45	23.7
DCGAN+CBAM	8.16	28.97	27.9
DCGAN+ECA	7.96	29.87	20.1
DCGAN+ReLU	6.85	38.52	18.5
DCGAN+GELU	7.92	30.18	22.6
DCGAN+SELU	8.62	24.53	19.0
ES-DCGAN	9.05	20.23	21.3

Compared with the baseline DCGAN, the ES-DCGAN improves the IS by 32.5% and reduces the FID by 47.7%, confirming the complementary benefits of integrating the ECA mechanism and the SELU activation function. Furthermore, compared with the other DCGAN variants (DCGAN+SE, DCGAN+CBAM, and DCGAN+GELU), the ECA mechanism yields slightly better performance than SE does and achieves competitive results comparable to those of the CBAM while maintaining lower computational latency. Moreover, the SELU activation function outperforms the GELU function in terms of improving the visual quality of the generated defect images.

## 5. Defect detection: The lightweight GE-YOLO11n model

### 5.1. Lightweight GhostConv module

In conventional feature extraction tasks, the standard convolution approach relies on a predefined kernel to perform sliding operations over the input feature map. Its computational complexity increases substantially under increased kernel sizes and channel dimensions, leading to higher overall model complexity. Moreover, the relatively linear feature extraction paradigm of the conventional convolution scheme restricts its ability to capture diverse and subtle feature patterns, which is particularly detrimental for steel surface defect detection tasks in which fine-grained textures are abundant. To address these limitations, the lightweight GhostConv module is incorporated into this paper [21]. GhostConv adopts a two-stage feature generation strategy: it first uses a standard convolution to produce a small set of intrinsic feature maps and then applies a series of inexpensive linear transformations to these intrinsic maps to rapidly generate multiple ghost feature maps. The intrinsic and ghost features are finally concatenated to maintain channel consistency with the standard convolution.

**Fig 11:**
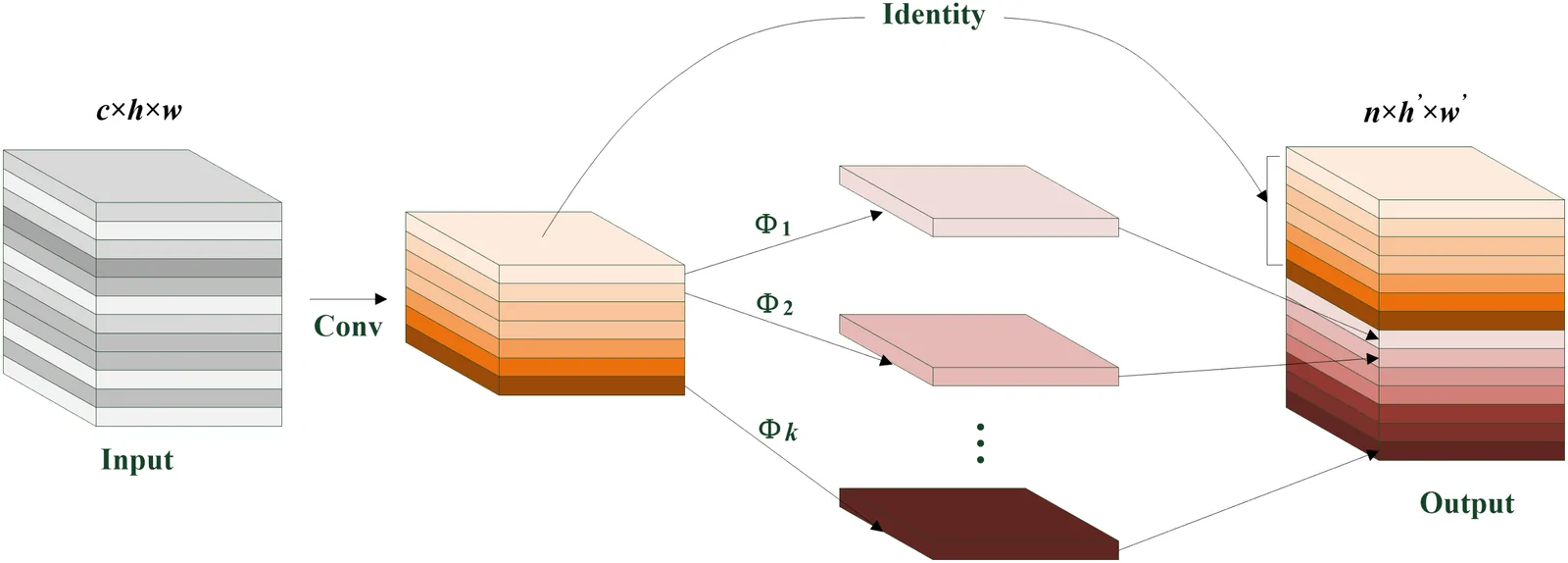
Structural diagram of GhostConv.

With respect to steel plate surface defect detection—which is characterized by significant scale variations and rich fine-grained details—GhostConv substantially reduces the number of model parameters while preserving the ability to extract subtle defect patterns. By leveraging inexpensive transformations to obtain diverse features from intrinsic maps, it achieves a favorable tradeoff between efficiency and representational capacity. Integrating GhostConv into the YOLO framework effectively decreases the computational overhead without sacrificing detection accuracy. The architecture of GhostConv is illustrated in Fig 11.

During the GhostConv process, it is assumed that the input feature map has dimensions of *c* × *h* × *w* and that the output feature map has dimensions of *n* × *h*^*’*^ × *w*^*’*^. The ratio of the computational quantities of ordinary convolution operations to those of GhostConv is as follows:


$$\begin{array}{c} r=\frac{n\cdot h^{\prime }\cdot w^{\prime }\cdot c\cdot k\cdot k}{\frac{n}{s}\cdot h^{\prime }\cdot w^{\prime }\cdot c\cdot k\cdot k+\left(s-1\right)\cdot \frac{n}{s}\cdot h^{\prime }\cdot w^{\prime }\cdot d\cdot d}\approx \frac{c\cdot s}{c+s-1}\approx s \end{array}$$ (15)

where *k* is the size of the regular convolution kernel, *n*/*s* is the number of output channels, *s* is the number of linear transformations (s<<*c*), and *d* is the size of the convolution kernel employed for linear transformations.

### 5.2. GE-YOLO11n model structure

**Fig 12:**
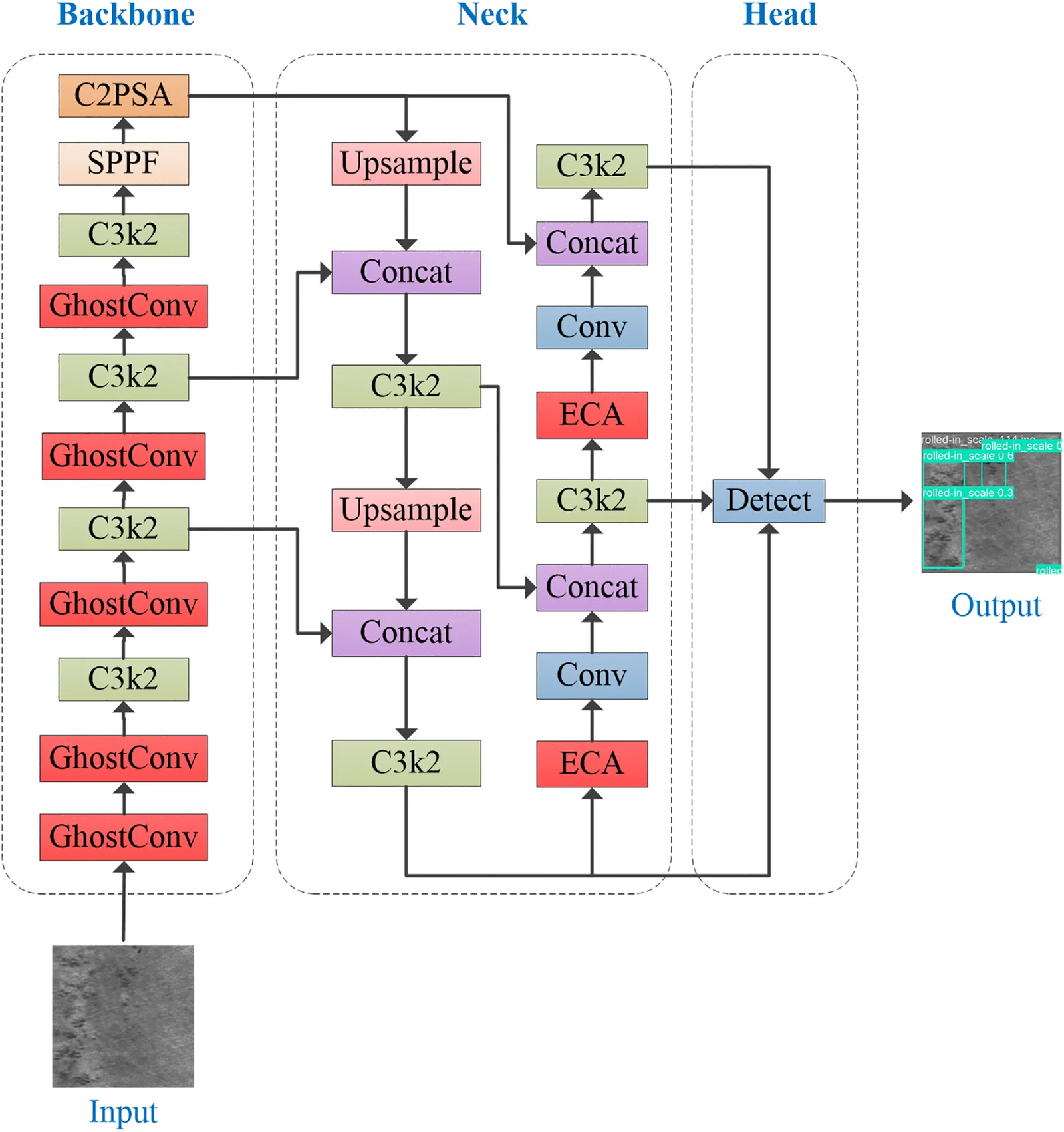
Model structure of GE-YOLO11n.

The YOLO11n network [22,23] comprises four core components: an input module, a backbone, a neck, and a detection head. The input module feeds steel plate defect images into the model; the backbone extracts hierarchical features from these images; the neck aggregates and fuses the multiscale feature maps acquired from the backbone; and the detection head, which is trained on annotated datasets, accurately performs defect localization and classification. However, in steel plate inspection scenarios, the inherent structural complexity of the original YOLO11n network incurs considerable computational overhead and increased latency, rendering it inadequate for industrial deployment scenarios. To overcome this limitation, we propose a lightweight variant, termed GE-YOLO11n, built upon the original framework. As illustrated in Fig 12, the lightweight GhostConv module is embedded into the backbone to replace the standard convolutions, effectively reducing the number of model parameters and the incurred computational cost. Moreover, the ECA mechanism is integrated into the neck to enhance the feature representations produced for small targets and defects of varying scales.

### 5.3. Evaluation indicators

(1) Precision *P* and recall *R*

The formulas for calculating *P* and *R* are as follows:


$$\begin{array}{c} P=\frac{TP}{TP+FP}=\frac{TP}{all\text{\textbackslash{}hspace\{0.33em}}detections\} \end{array}$$ (16)

$$\begin{array}{c} R=\frac{TP}{TP+FN}=\frac{TP}{all\text{\textbackslash{}hspace\{0.33em}}ground\text{\textbackslash{}hspace\{0.33em}\}truths\} \end{array}$$ (17)

where a *TP* denotes a correct detection with an IOU exceeding the threshold, an *FP* denotes an incorrect detection with an IOU below the threshold, an *FN* means that the ground truth is not detected, and a *TN* means that no ground truth is present and remains undetected.

(2) Average precision and mean average precision

Among a total of *N* samples, there are *M* positive samples. After all the samples are sorted by their confidence levels, we can obtain *M* recall values corresponding to the Top-1 to Top-*M* entries. For each recall value, we can obtain the maximum precision *P* when the corresponding recall is greater than or equal to the current recall value. Calculating the average of these *M* values yields the average precision (AP) metric. The mean average precision (mAP) is then obtained by computing the mean of the AP values calculated across all classes.

(3) Latency per image and FPS (frame rate)

The formula for calculating the latency per image is as follows:


$$\begin{array}{c} t=\frac{T}{N} \end{array}$$ (18)

The FPS calculation formula is as follows:


$$\begin{array}{c} t^{\prime }=\frac{N}{T} \end{array}$$ (19)

where *T* represents the total latency and *N* represents the number of images.

## 6. Analysis of the experimental results

### 6.1. Testing environment and datasets

The experiments conducted in this paper are run under the PyTorch framework, and the employed configurations are shown in Table 6.

**Table 6:** Configuration information of the experimental environment.

Attribute	Configuration
System	Ubuntu 18.04, 64-bit version
CPU	Intel Core i7-7700K
GPU	NVIDIA GeForce GTX 1080 Ti, 11 GB
Language	Python 3.9
DL framework	PyTorch 2.1.0

The original NEU-DET dataset contains 1800 images, which are split into an original training set (1260), a validation set (360), and a test set (180) at a ratio of 7:2:1. Subsequently, only the 1260 original training images are used for sample generation purposes, expanding the total number of samples to 3060 (1260 original training images and 1800 generated training images). Notably, all the generated samples are exclusively included in the training set and do not appear in the validation set or the test set. For the generated samples, a manual annotation process is performed as follows.

(1) **Annotators and their professional qualifications:** The three annotators involved in the labeling work are all senior industrial defect detection engineers from the China Heavy Machinery Research Institute, each with more than five years of practical experience in steel plate surface defect identification and industrial image annotation; they are also familiar with the formation mechanisms and visual characteristics of various rolling defects. A chief senior engineer from the institute serves as the final review expert and is specifically responsible for the ultimate evaluations produced for ambiguous samples and low-quality samples.
(2) **Labeling process:** All the images are uniformly annotated using the LabelImg tool, with a prescreening and multilevel verification mechanism implemented. 1) Prior to the annotation step: Low-quality images, including invalid samples with severely blurred defect features, exposure imbalances, or noise completely obscuring their defect contours, are excluded. 2) Independent annotation: Each image is separately annotated by three engineers regarding their defect locations and categories. If at least two annotators agree on the defect classification, the image is assigned to the corresponding class. 3) Final review of ambiguous samples: if the annotations obtained from all three engineers differ, the sample is deemed ambiguous and referred to the chief senior engineer for re-evaluation. If an annotation can be made based on industrial defect standards, the sample is retained; if the defect class remains unresolved after the final review, the sample is directly discarded.

**Fig 13:**
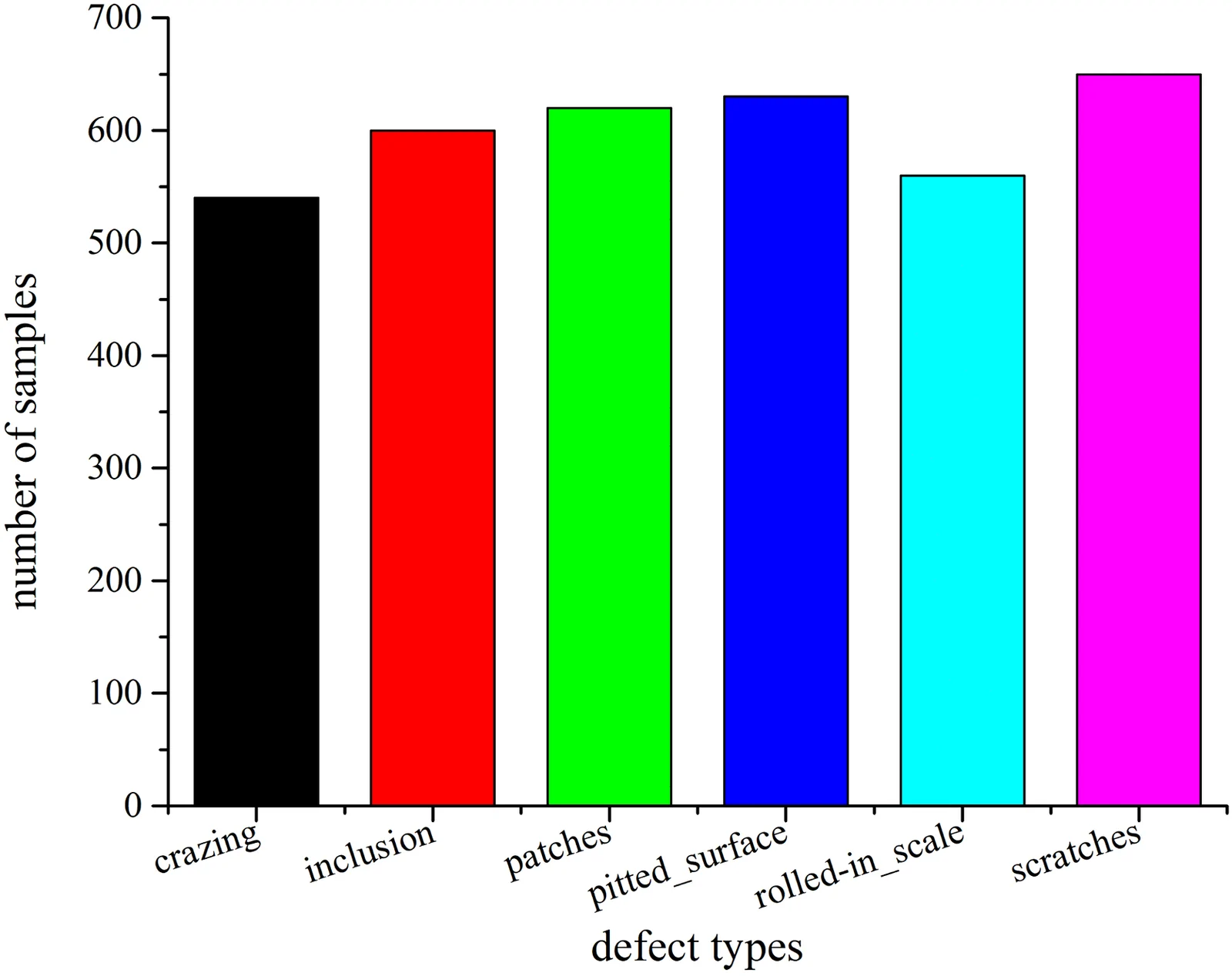
Numbers of samples for six classes of defects.

In conclusion, the new dataset constructed in this paper includes 3060 training images, 360 validation images, and 180 test images. The samples collected for each of the six classes are presented in Fig 13, which contains 540 crazing images (450 training images+60 validation images+30 test images), 600 inclusion images (510 training images+60 validation images+30 test images), 620 patches (530 training images+60 validation images+30 test images), 630 pitted_surface images (540 training images+60 validation images+30 test images), 560 rolled-in_scale images (470 training images+60 validation images+30 test images) and 650 scratches (560 training images+60 validation images+30 test images).

### 6.2. YOLO model optimization based on the original NEU-DET dataset

(1) Parameter optimization of the baseline model based on the NEU-DET

The YOLO11n and YOLO11m baseline models are applied to the original NEU-DET dataset (1260 training images+360 validation images+180 test images). The detection results obtained for the validation images are shown in Table 7. The optimal performance is achieved when the number of epochs is set to 200 and the batch size is 16. Although YOLO11m achieves a 0.2% improvement in accuracy relative to YOLO11n, its model parameters are approximately 7 times those of YOLO11n in number, and its detector-only latency is approximately 4 times that of YOLO11n. Considering the balance between detection accuracy and model weight, YOLO11n is selected as the baseline model in this paper.

**Table 7:** Detection results obtained on the validation images under different parameter settings.

Model	Number of epochs	batch_size	mAP@.5 (%)	Params (M)	Detector-only latency (ms)
YOLO11n	100	32	84.1	2.8	5.2
YOLO11n	200	16	85.3		
YOLO11m	100	32	83.2	21.6	22
YOLO11m	200	16	85.5

(2) Structural optimization of the YOLO model based on NEU-DET

Starting from the baseline model, the GhostConv, ECA, and GhostConv+ECA (GE-YOLO11n) modules are sequentially integrated, with the hyperparameters set as epoch = 200 and batch_size = 16. These modified models are also applied to the original NEU-DET dataset (1260 training images+360 validation images+180 test images), and a comparison among the parameters of the validation images produced for different YOLO variants is shown in Table 8. The proposed GE-YOLO11n model achieves an mAP of 86.8%, a parameter count of 2.1 M, and a detector-only latency of 4.9 ms. Although its mAP is 0.5% lower than that of YOLO11n+ECA, the detector-only latency is reduced by 0.5 ms, and the number of parameters is reduced by 0.8 M. While its detector-only latency is 0.1 ms greater than that of YOLO11n+GhostConv, the mAP is improved by 2.2%. It achieves an optimal balance between accuracy and efficiency.

**Table 8:** Comparison among the parameters of the validation images produced for different YOLO variants.

Model	P (%)	R (%)	F1 (%)	mAP@.5 (%)	mAP@.5:.95 (%)	Params (M)	Detector-only latency (ms)
YOLO11n	87.5	84.2	85.8	85.3	61.4	2.8	5.2
YOLO11n+GhostConv	86.1	82.5	84.3	84.6	60.9	2.0	4.8
YOLO11n+ECA	89.2	85.8	87.5	87.3	62.7	2.9	5.4
GE-YOLO11n	88.6	85.3	86.9	86.8	62.3	2.1	4.9

### 6.3. Analysis of ablation experiments

**Fig 14:**
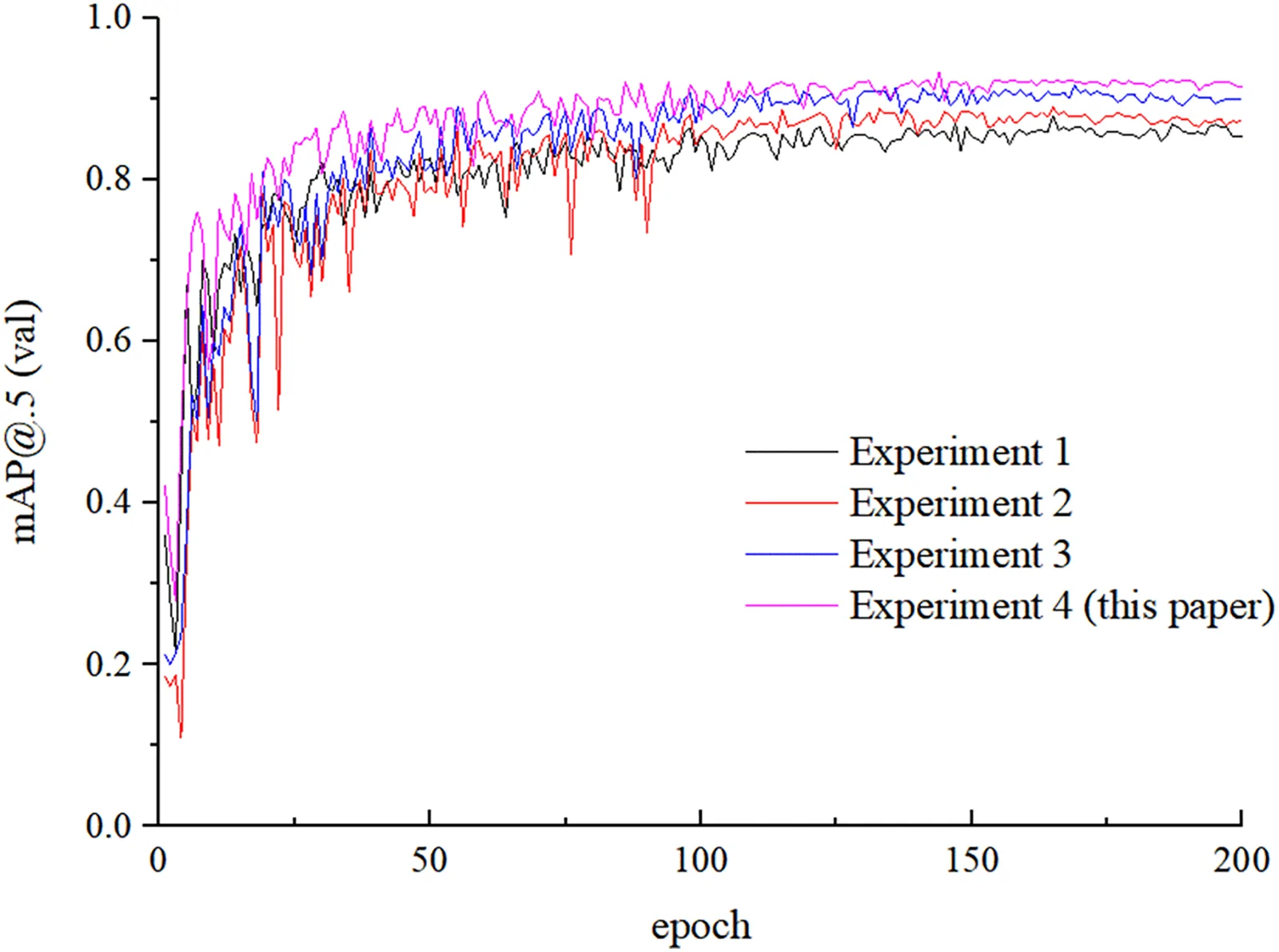
mAPs achieved on the validation images obtained for the four experiments.

Four sets of ablation experiments are designed; the datasets and models adopted in each experiment are presented in Table 9, with the other parameters kept consistent. The mAP values obtained on the validation images of the four experiments are shown in Fig 14. Experiment 4 (the approach of this paper) yields the highest mAP value, with an optimal performance level of 91.6%. A detailed comparison among the results of the ablation experiments conducted on the validation images is shown in Table 10, where the full-pipeline latency includes preprocessing, resizing, model inference, nonmaximum suppression, and postprocessing. Upon introducing the ES-DCGAN, AW-ACE and GE-YOLO11n modules, the overall mAP is improved by 6.3%, and the number of parameters is reduced by 21.4%, which reflects the cumulative effect of the tested pipeline. While the full-pipeline latency increases by 0.5 ms, the proposed method effectively balances accuracy and efficiency.

**Table 9:** The datasets used in the four experiments.

Number	Dataset	Sample size	Sample split	Model
Experiment 1	NEU-DET	1800	1260 training images+360 validation images+180 test images	YOLO11n
Experiment 2	NEU-DET processed by AW-ACE	1800	1260 training images+360 validation images+180 test images	YOLO11n
Experiment 3	NEU-DET processed by the ES-DCGAN and AW-ACE	3600	3060 training images+360 validation images+180 test images	YOLO11n
Experiment 4	NEU-DET processed by the ES-DCGAN and AW-ACE	3600	3060 training images+360 validation images+180 test images	GE-YOLO11n

**Table 10:** Comparison among the four experiments in terms of their validation images.

Experiment	mAP@.5 (%)	Params (M)	Full-pipeline latency (ms)
Experiment 1	85.3	2.8	6.7
Experiment 2	87.5	2.8	7.5
Experiment 3	90.2	2.8	7.5
Experiment 4 (this paper)	91.6 (↑6.3)	2.2	7.2

### 6.4. GE-YOLO11n detection results

**Fig 15:**
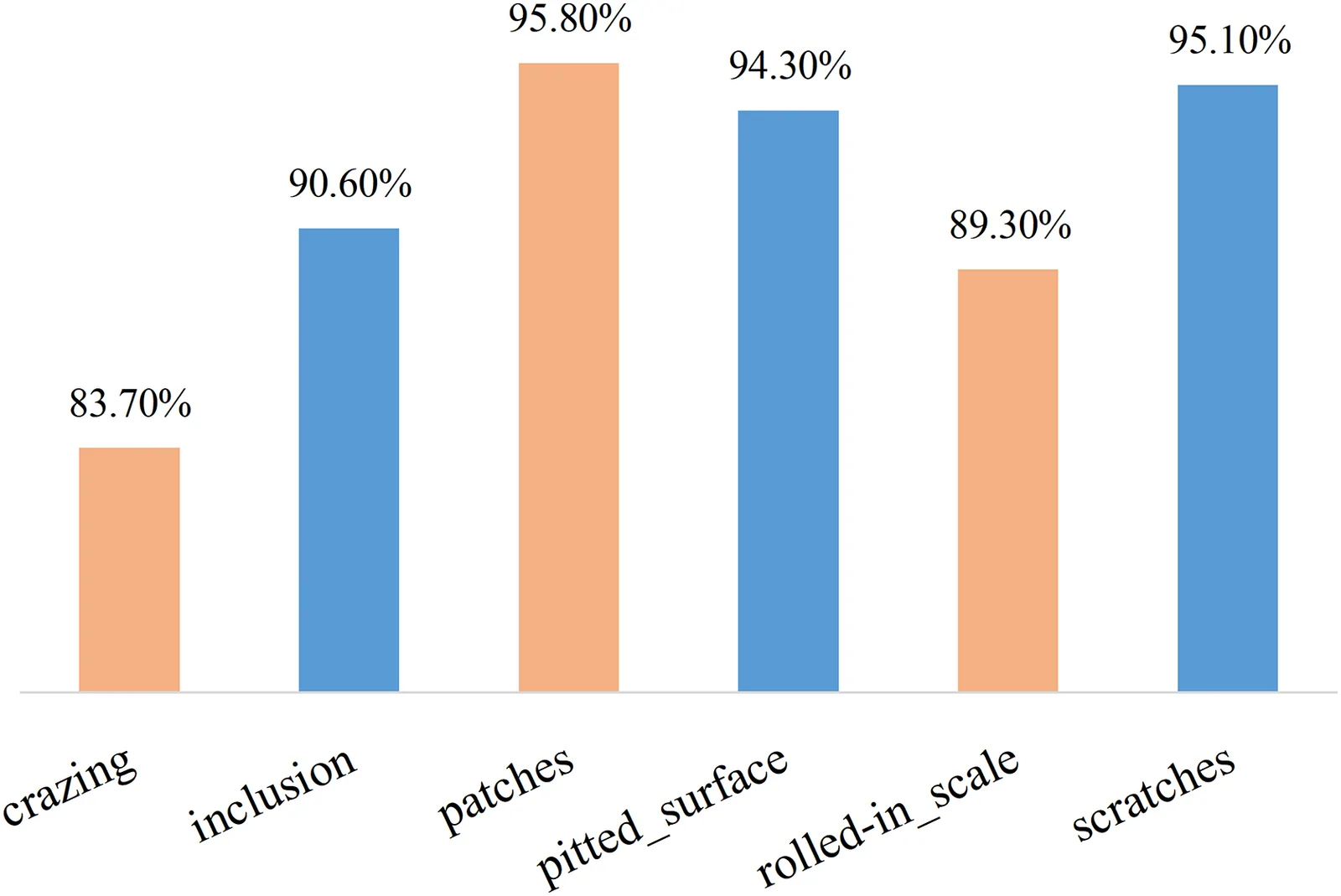
The mAP values produced on test images for six classes of defects.

**Fig 16:**
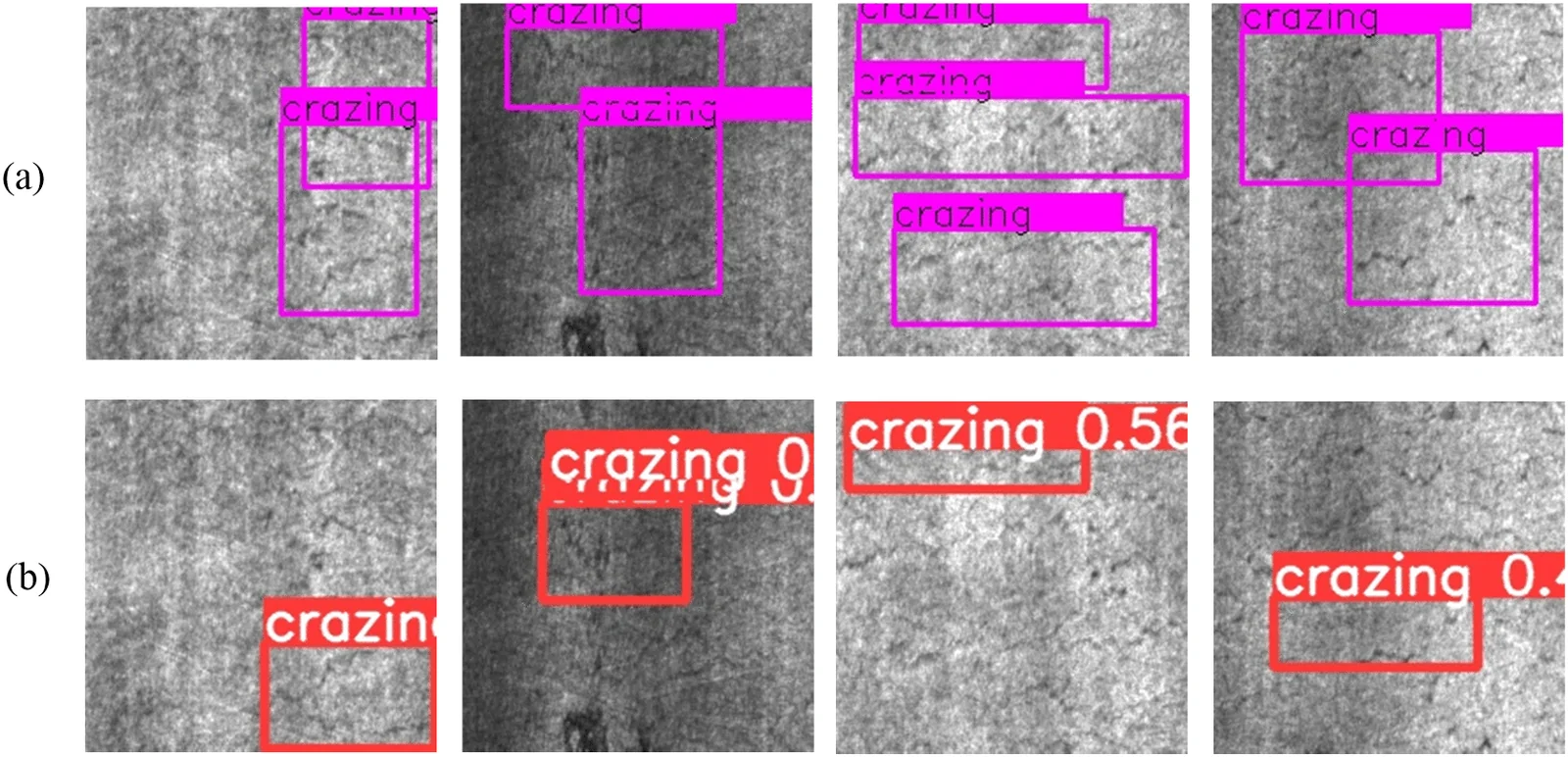
Visualization of the failure cases observed when conducting crazing defect detection on the test images. (a) Annotation results. (b) Detection results.

The GE-YOLO11n model is applied to the new NEU-DET dataset processed by AW-ACE and the ES-DCGAN (3060 training images+360 validation images+180 test images). The mAP values achieved in a single run on test images of the six defect types are presented in Fig 15, among which the mAP attained for patches is the highest (95.8%), while that observed for crazing is the lowest (83.7%). Some failure cases noted when conducting crazing defect detection on the test images are shown in Fig 16, where part of the crazing is misidentified as background information. Compared with other defect types, crazing has a smaller pixel size and a grayscale similar to that of the background. Owing to the low contrast and poor recognizability of crazing defects, it is difficult to extract effective features from them. On the other hand, densely intertwined cracks form a mesh-like structure that is accompanied by irregular annotation boxes, which makes it challenging for the feature fusion module to separate dense features. Consequently, crazing defects are prone to missed detections or misdiagnoses.

**Fig 17:**
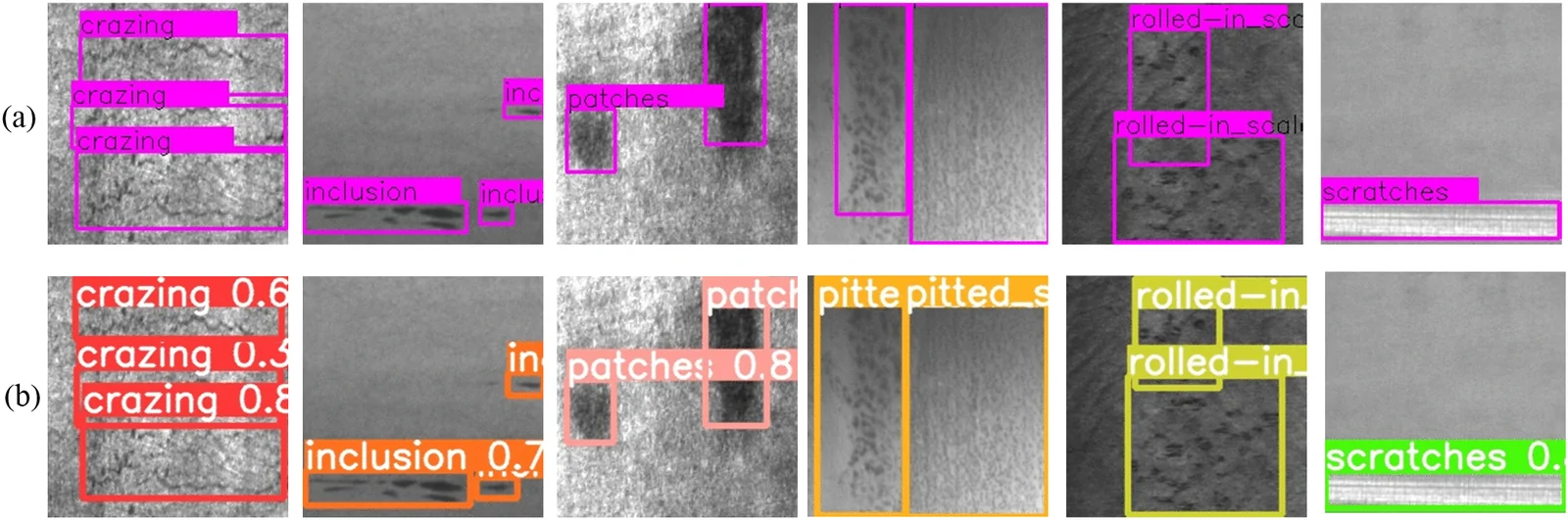
Visual comparison between the annotation and detection results produced on test images for six classes of defects. (a) Annotation results. (b) Detection results.

The detection results yielded by the proposed model on test images for the six defects are shown in Fig 17. The first row shows the annotation results, while the second row shows the detection results obtained by the proposed model. The detection results are essentially consistent with the manually annotated results, indicating that the proposed model can achieve accurate positioning and classification recognition for steel plate defects.

**Fig 18:**
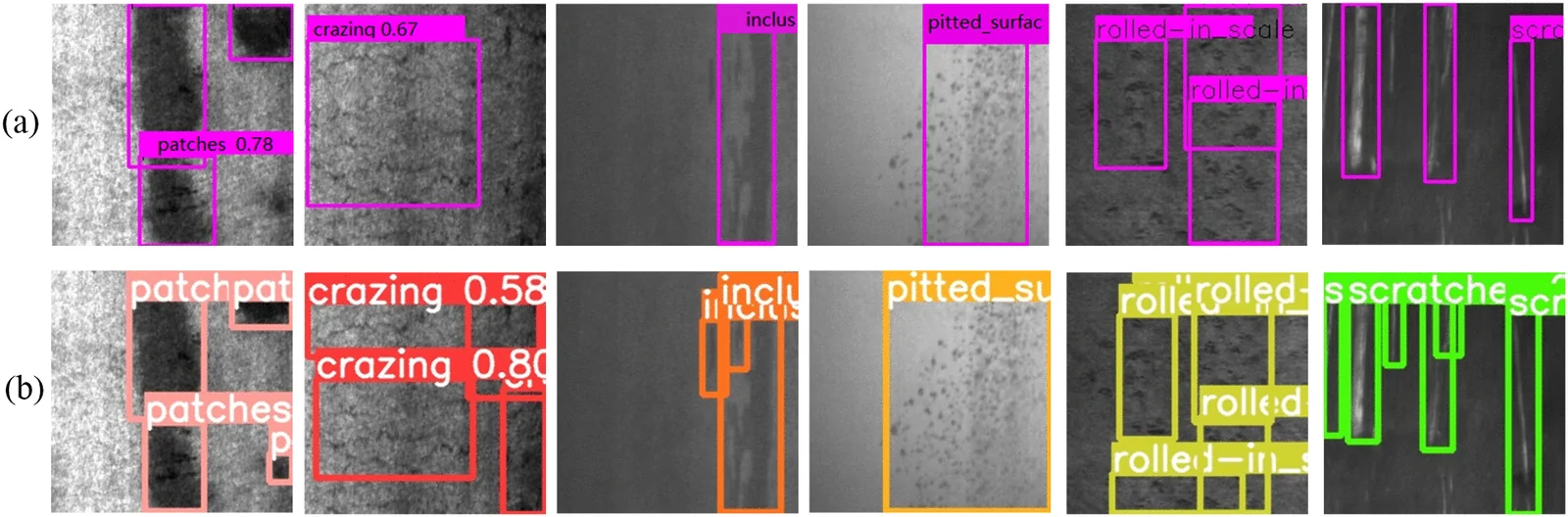
Comparison between the visual results produced on the test images before and after applying the YOLO11 improvement. (a) Detection results of YOLO11n. (b) Detection results of GE-YOLO11n.

A comparison between the visual detection results produced by the GE-YOLO11n and original YOLO11n models on the test images is shown in Fig 18, where both models are applied to the new NEU-DET dataset processed by AW-ACE and the ES-DCGAN (3060 training images+360 validation images+180 test images). The original YOLO11n model fails to detect defects such as crazing, patches, and scratches. In contrast, the proposed GE-YOLO11n model can detect more defect areas and demonstrates superior detection performance.

**Fig 19:**
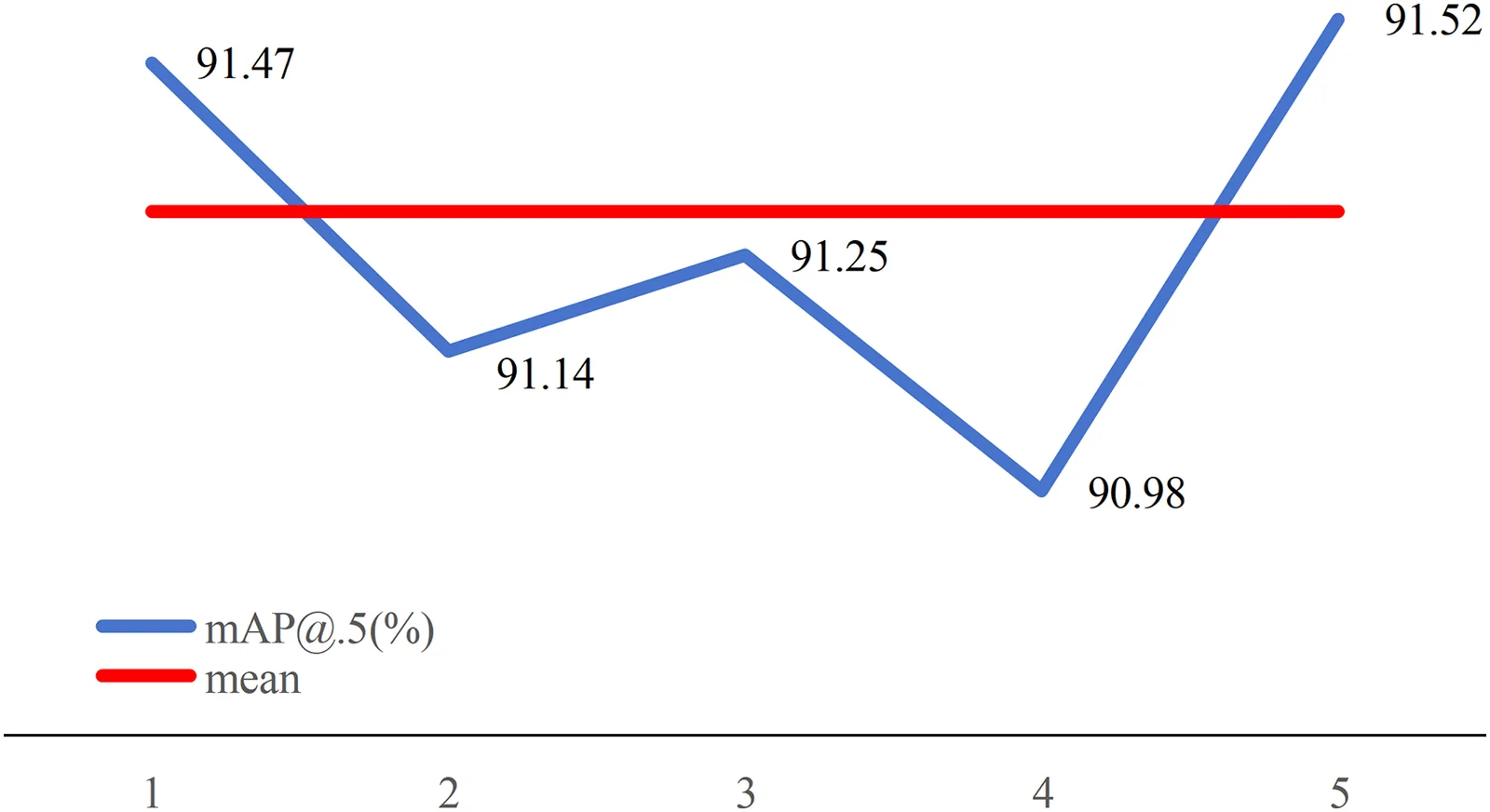
mAP and mean values of the five test results obtained on the test images.

Since a DCGAN is used in this paper to augment the training set, while the validation and test sets contain only real images, a cross-validation implemented between the training and validation sets would result in the images generated from the training set being mixed into the validation set. Therefore, a repeated random subsampling validation method is adopted. The 540 real defect images are randomly split into a validation set (360 images) and a test set (180 images) at a 2:1 ratio, with the training set remaining unchanged (containing both real and DCGAN-generated images). The performance of the model is validated on the test set. The random-split experiments are repeated five times. For each repetition, the detection model is independently trained, and its mAP@.5 is calculated on the corresponding test set. The mean and standard deviation calculated over five tests are reported to mitigate performance fluctuations caused by the use of a single data split. The mAP and mean values of the five tests are shown in Fig 19. The mean mAP@.5 value is 91.3%, with a standard deviation of 0.2%, so the result obtained from the five tests for mAP@0.5 is 91.3% ± 0.2%.

### 6.5. Comparison with other models

To further verify the superiority of the proposed model, Faster R-CNN [24,25], models of the YOLO series [26–28], and other lightweight defect detection models [29,30] are employed to perform detection on the datasets used in this paper. All the models use the NEU-DET dataset processed by AW-ACE and the ES-DCGAN (3060 training images+360 validation images+180 test images). The number of training epochs is set to 200, with a batch size of 16. For the YOLO series, the data augmentation process employs a fully consistent pipeline (including random horizontal flipping, random scaling, random cropping, and mosaic enhancement); mosaic enhancement is not applied to the non-YOLO-series models. In the postprocessing stage, the confidence threshold is set to 0.25, and the NMS threshold is set to 0.45. All the model metrics—including mAP@0.5, mAP@0.5:0.95, precision (P), and recall (R)—are calculated using standard methods. On this basis, the specific implementations and training configurations of each model are as follows. Faster R-CNN and RetinaNet employ the official ResNet50-FPN implementation, with parameter counts of 32.4 M and 37.8 M, respectively; both use the SGD optimizer at an initial learning rate of 0.01 without additional tuning. The Swin transformer (Swin-Tiny version), DETR (ResNet50 version), RT-DETR (R18), and FC-DETR (R18) all utilize their official implementations with parameter counts of 28.3 M, 41.4 M, 20.0 M, and 21.5 M, respectively; they uniformly employ the AdamW optimizer at an initial learning rate of 1e-4 without additional structural modifications. Lightweight YOLOv5l, YOLOv8n, YOLOv8n-CSG, EfficientDet-D0, Steel-YOLOv8n, and DCN-YOLOv8n all use the SGD optimizer at an initial learning rate of 0.01, with parameter counts ranging from 2.3 to 26 M. The GE-YOLO11n model proposed in this study maintains identical hyperparameters (including the optimizer and learning rate) to its corresponding native counterparts, with only targeted modifications applied to the neck/backbone architecture. The detailed performance of each model is shown in Table 11, which displays the mean values of the five test results, where the full-pipeline latency includes preprocessing, resizing, model inference, nonmaximum suppression, and postprocessing steps. The experimental results show that the proposed model achieves a precision rate (P) of 91.5%, a mean average precision rate (mAP@.5) of 91.3%, and a full-pipeline latency level of 7.4 ms. The key observations are as follows. (1) Accuracy: The proposed GE-YOLO11n network achieves 91.3% mAP@0.5, thus outperforming all the non-YOLO methods, such as RT-DETR, FC-DETR and the Swin transformer. It also outperforms the lightweight YOLOV5l model. (2) Full-pipeline latency: GE-YOLO11n runs at 7.4 ms, which is significantly faster than all the non-YOLO methods. Even among the YOLO variants, it achieves the lowest latency. (3) Overall: The proposed GE-YOLO11n network achieves the highest mAP@0.5 value of 91.3% and the highest mAP@0.5:0.95 value of 66.9% among all the compared methods. In terms of full-pipeline latency, compared with all the non-YOLO models, GE-YOLO11n runs at 7.4 ms per image, making it the fastest method. Even among the YOLO variants, GE-YOLO11n offers the best accuracy-efficiency tradeoff.

In short, the proposed method consistently achieves the best tradeoff between detection accuracy and efficiency, regardless of whether it is compared with YOLO-based or non-YOLO state-of-the-art detectors. Notably, this comparison involves experimental configuration differences: only the YOLO models utilize the mosaic enhancement process, whereas the non-YOLO models do not employ this strategy; most baseline models use the official configurations without undergoing equivalent global parameter tuning work. Therefore, the performance improvement observed in this study applies solely to the current experimental setup and does not reflect the absolute performance of the developed model under unified optimal parameter tuning and identical enhancement strategies.

**Table 11:** Comparison among different detection models.

Model	P (%)	R (%)	mAP@.5 (%)	mAP@.5:.95 (%)	Full-pipeline latency (ms)
Faster R-CNN (ResNet50)	72.9	70.7	72.3	46.8	63.8
RetinaNet (ResNet50)	79.2	77.5	77.9	54.3	45.1
Swin Transformer (Swin-Tiny)	82.7	80.5	82.3	55.7	29.2
DETR (ResNet50)	75.8	73.2	73.9	49.2	49.6
RT-DETR (R18)	85.2	83.7	84.3	60.1	20.7
FC-DETR (R18)	86.1	84.3	84.5	61.5	21.4
Lightweight YOLOV5l	91.1	89.2	90.4	64.5	27.8
YOLOv8n	83.1	82.3	83.7	59.8	10.2
YOLOv8n-CSG	86.3	84.5	85.2	60.7	8.6
EfficientDet-D0	86.4	85.9	85.5	62.4	12.3
Steel-YOLOv8n	89.9	87.2	88.4	63.8	10.8
DCN-YOLOv8n	86.7	85.6	86.5	61.2	12.3
YOLO11n	89.8	87.7	90.6	66.2	7.9
GE-YOLO11n	91.5	89.3	91.3	66.9	7.4

### 6.6. Validation conducted on the GC10-DET dataset

The GC10-DET dataset includes 10 defect classes. The original GC10-DET dataset contains three types of issues: unlabeled samples, label spelling errors, and inconsistent naming conventions for defects within the same class. The data correction process involves the following steps. (1) Removal of unlabeled and duplicate images: Unlabeled images: img_08_4406743300_00407, img_05_425382900_00002, img_06_425614600_0042, img_06_425614600_00438, img_07_3436814000_00020, and img_07_436164700_01539. Duplicate images: img_03_425506300_00018, img_06_425502900_00052, img_07_425391800_00054, img_07_425502900_00052, img_04_425503600_00017, img_06_425505500_00052, img_07_436163600_01161, img_01_425503100_00018, img_07_425503000_00061, img_02_425392000_00984, img_03_436068500_00002, and img_06_3436814000_00687. (2) Correction of erroneous labels: Replacing “d” in the label file titled img_02_425616500_00770 with “1_chongkong”. (3) Standardization of similar labels: Replacing “10_yaozhed” in the 131 label files with “10_yaozhe”. The pseudocode implementation is shown in Table 12.

**Table 12:** Algorithm for GC10-DET annotation label standardization.

Algorithm: GC10-DET Annotation Label Standardization
**Input**: Root directory path *base_dir*, invalid category label *old_class*, standardized label *new_class* **Output**: Count of modified annotation files *file_count*, total corrected object instances *obj_count* 1. *label_dir*←JoinPath(*base_dir*,“lable”) 2. Initialize *file_count*←0, *obj_count*←0 3. **if** PathExists(*label_dir*)=False **then** 4. Print warning: “Label folder does not exist” 5. Terminate algorithm 6. **for each** annotation file *xml_file* in ListDir(*label_dir*) **do** 7. **if** suffix of *xml_file ≠* “.xml” **then** skip current file 8. *xml_path*←JoinPath(*label_dir*,*xml_file*) 9. **try**: 10. *tree*←ParseXML(*xml_path*) 11. *root*←GetRoot(*tree*) 12. *modified*←False 13. **for each** object node obj in FindAll(*root*,“object”) **do** 14. *name_tag*←FindChild(*obj*,“name”) 15. **if** *name_tag ≠* Null and Text(*name_tag*)=*old_class* **then** 16. SetText(*name_tag*,*new_class*) 17. *obj_count*←*obj_count*+1 18. *modified*←True 19. **if** *modified* = True **then** 20. SaveXML(*tree*,*xml_path*,encoding = utf-8) 21. *file_count*←*file_count*+1 22. Print modified file name *xml_file* 23. **catch parsing error**: Skip corrupted annotation file 24. Return *file_count*, *obj_count*

**Fig 20:**
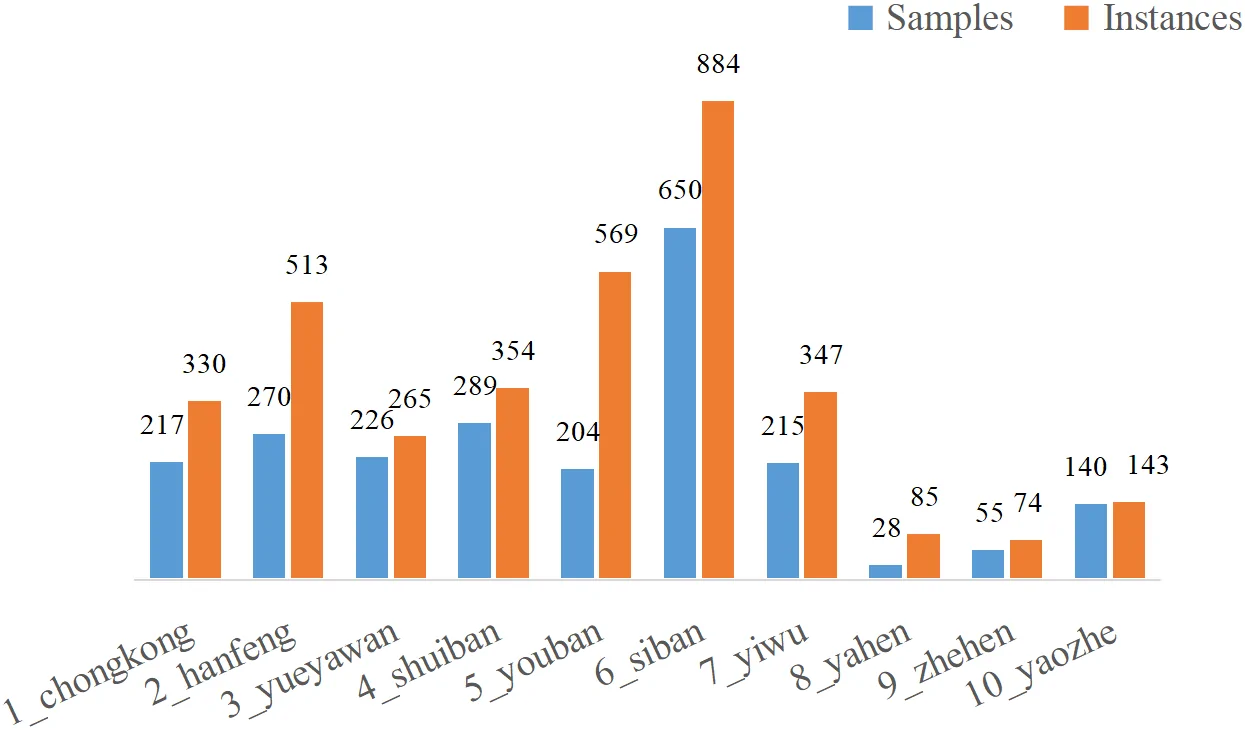
Samples and instances of each class.

After applying the aforementioned corrections, a total of 2294 images and 3564 instances are obtained (some images contain multiple defect instances) for the subsequent model experiments. The English translations of the ten defect classes are shown in Table 13, and the numbers of samples and instances for the ten classes are shown in Fig 20.

**Table 13:** English translations of the ten defect classes.

Label	1_chongkong	2_hanfeng	3_yueyawan	4_shuiban	5_youban
**English translation**	punching_hole	welding_line	crescent_gap	water_spot	oil_spot
**Label**	6_siban	7_yiwu	8_yahen	9_zhehen	10_yaozhe
**English translation**	silk_spot	inclusion	rolled_pit	crease	waist folding

After the GC10-DET dataset is processed with the proposed AW-ACE image enhancement module, the data are divided into a training set, a validation set, and a test set at a ratio of 7:2:1. The trained GE-YOLO11n model (epoch = 200) is applied to the test images, and the parameters for the ten classes of defects that are identified in a single run are shown in Table 14. The overall P value is 91.5%, the R is 88.9%, the mAP@.5 is 76.1%, and the mAP@.5:.95 is 54.3%, demonstrating excellent detection performance. Among these outcomes, three defect classes—1_chongkong, 3_yueyawan and 10_yaozhe—yield the best results, with both the precision and recall values exceeding 93% and the mAP@.5 surpassing 86%. This is primarily because these defect classes have clear boundaries, regular shapes, and high levels of contrast with the background, which facilitates the ability of the model to learn stable feature patterns. In contrast, three classes—7_yiwu, 8_yahen and 9_zhehen—yield lower performance. This is because these defects typically exhibit low contrast levels, blurred boundaries, and variable shapes, making them susceptible to interference from background noise. This increases the difficulty faced by the model when attempting to learn effective features, resulting in higher rates of missed and false detections.

**Table 14:** Parameters of the ten defect classes.

Class	P (%)	R (%)	F1 (%)	mAP@.5 (%)	mAP@.5:.95 (%)
1_chongkong	96.5	94.5	95.5	89.2	72.5
2_hanfeng	93.2	91.8	92.5	83.4	65.2
3_yueyawan	95.8	93.7	94.7	86.3	71.7
4_shuiban	91.7	87.9	89.8	70.9	48.8
5_youban	92.3	89.6	90.9	80.7	55.9
6_siban	92.1	90.8	91.4	81.9	51.8
7_yiwu	86.3	81.6	83.9	52.9	35.4
8_yahen	88.2	85.3	86.7	58.3	31.6
9_zhehen	83.6	80.4	82.0	62.8	37.9
10_yaozhe	95.4	93.1	94.2	94.9	72.2
All	91.5	88.9	90.2	76.1	54.3

**Fig 21:**
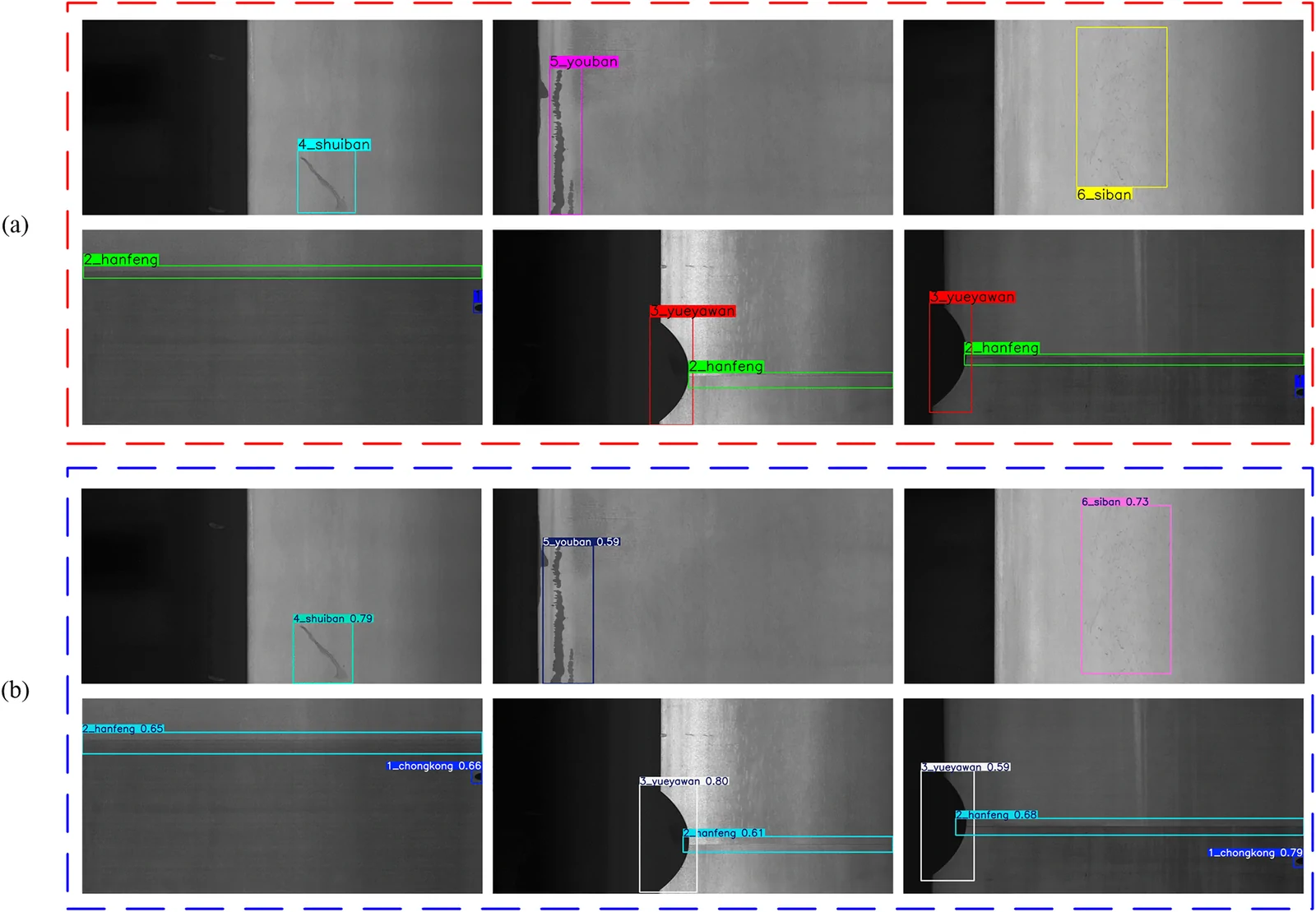
Visual detection results produced by GE-YOLO11n on the test images. (a) Annotation results. (b) Detection results.

**Fig 22:**
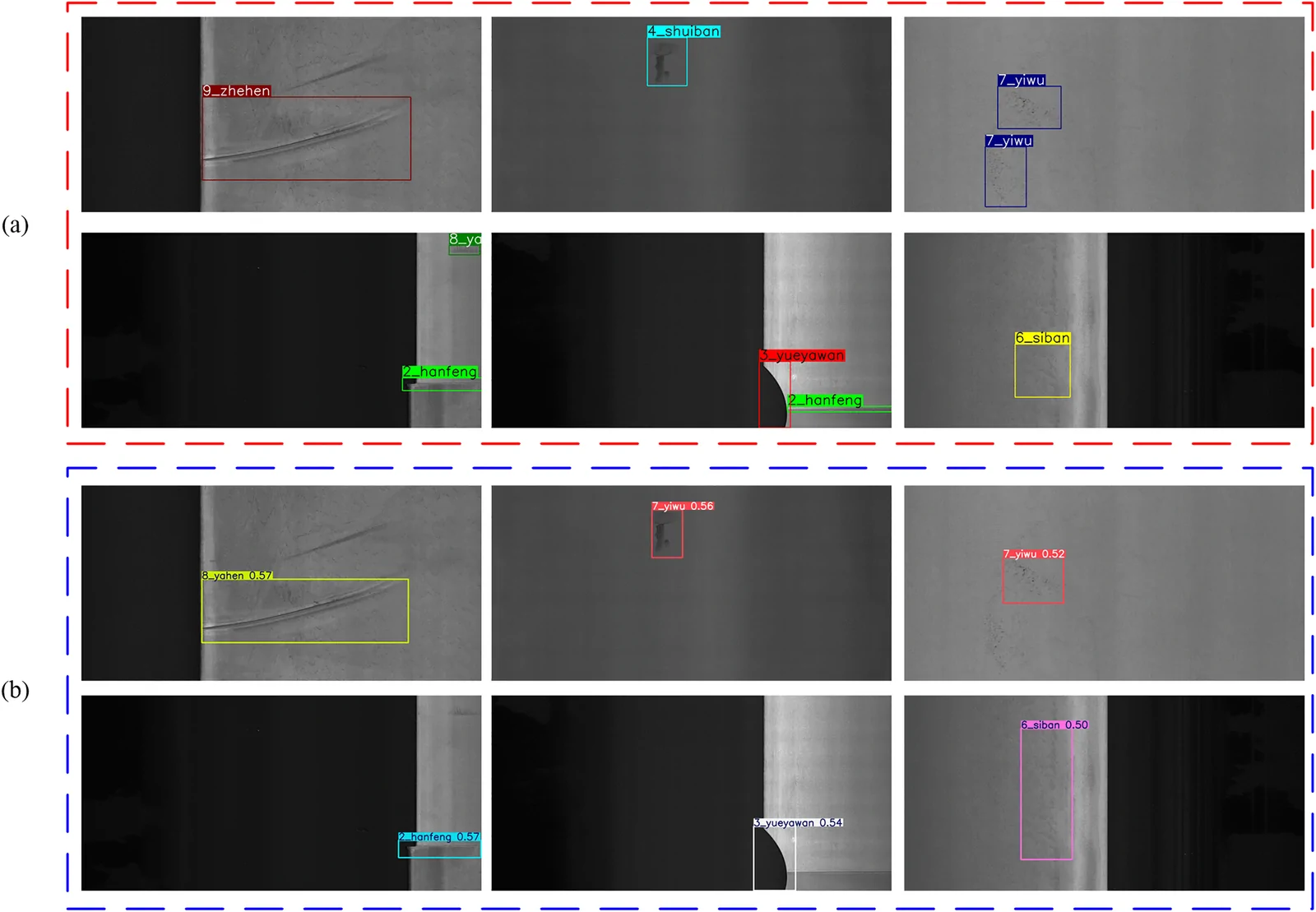
Visualization of the failure cases observed when performing defect detection on the test images. (a) Annotation results. (b) Detection results.

Some visual detection results produced on the test images are shown in Fig 21, where both single and multiple targets in the images are accurately detected, and their corresponding classes are correctly identified. Some failure detection cases are shown in Fig 22, and the detection errors primarily manifest in three typical issues: missed detections, misclassifications, and detection box deviations. A detailed analysis is provided as follows.

(1) Missed detections: In the annotations obtained for the left image in the second row, both 8_yahen and 2_hanfeng defects are present; however, the model identifies only 2_hanfeng in the corresponding detection results, omitting 8_yahen. 8_yahen is a small-scale, low-contrast defect with weak visual features in the image, making it susceptible to interference from background noise or other defects, thus preventing the model from effectively capturing this target.
(2) Misidentifications: The left image in the first row is labeled 9_zhehen but is misidentified as 8_yahen in the detection results. Among the two “7_yiwu” labels in the right image of the first row, while the model identifies the correctly class, its confidence score is low. Both 9_zhehen and 8_yahen exhibit similar morphological features—linear or locally depressed patterns—which can lead to confusion during the feature extraction procedure. Additionally, the defect boundaries of “7_yiwu” are blurred, and its features are inconspicuous, resulting in poor distinguishability from the background or other defects and, consequently, insufficient model confidence.
(3) Detection box deviations: The annotation box produced for the 6_siban defect in the right image of the second row shows a significant positional deviation from the detection box. Given that the 6_siban defect itself exhibits weak features and closely resembles the background texture, the model struggles to accurately locate its center, resulting in detection box displacement.

A comparison between YOLO11n and GE-YOLO11n is shown in Table 15. All parameters are the mean values of the five test results, and the full-pipeline latency includes preprocessing, resizing, model inference, nonmaximum suppression, and postprocessing steps. Compared with the original YOLO11n model, the GE-YOLO11n model achieves a 2.8% increase in precision (P), a 3.1% increase in mAP@.5, and a 3.5 ms reduction in full-pipeline latency.

**Table 15:** Comparison between YOLO11n and GE-YOLO11n.

Model	P (%)	mAP@.5 (%)	mAP@.5:.95 (%)	Full-pipeline latency (ms)
YOLO11n	88.4	72.8	52.5	34.6
GE-YOLO11n	91.2	75.9	54.2	31.1

## 7. Conclusion

An improved YOLO11n-based steel plate defect detection method is proposed. AW-ACE is proposed to enhance defect features, an ES-DCGAN is designed to increase the attention paid by the generation network to different features and expand defect samples, and GE-YOLO11n is introduced to improve the feature extraction ability of the method for small targets. Ablation studies demonstrate that the complete pipeline, comprising the ES-DCGAN, AW-ACE, and GE-YOLO11n modules, improves the overall mAP by 6.3% and reduces the number of required parameters by 21.4%. Compared with Faster R-CNN, DETR, and the YOLO series and variants, the method developed in this paper achieves 91.3% mAP and 7.4 ms full-pipeline latency, outperforming the other compared models under the experimental conditions specified in this study.

This study faces challenges in terms of quantitatively determining whether minor precision improvements constitute statistically significant differences. In future research, we will increase the number of experimental repetitions and incorporate statistical measures such as standard deviations and confidence intervals to enhance the uncertainty analysis. Additionally, we will standardize the enhancement strategy and parameter tuning process to ensure complete fairness in any comparisons conducted in our subsequent work.
